# Supplementary material for: Structural basis of promiscuous substrate transport by Organic Cation Transporter 1
Source: Nat Commun. 2023 Oct 11;14:6374. doi: 10.1038/s41467-023-42086-9 (PMC10567722; doi:10.1038/s41467-023-42086-9)
Supplement: Supplementary file 1 — Supplementary Information [file 41467_2023_42086_MOESM1_ESM.pdf]

Supplementary Information

Structural Basis of Promiscuous Substrate Transport in Organic Cation Transporter 1

Yi C Zeng, et al.

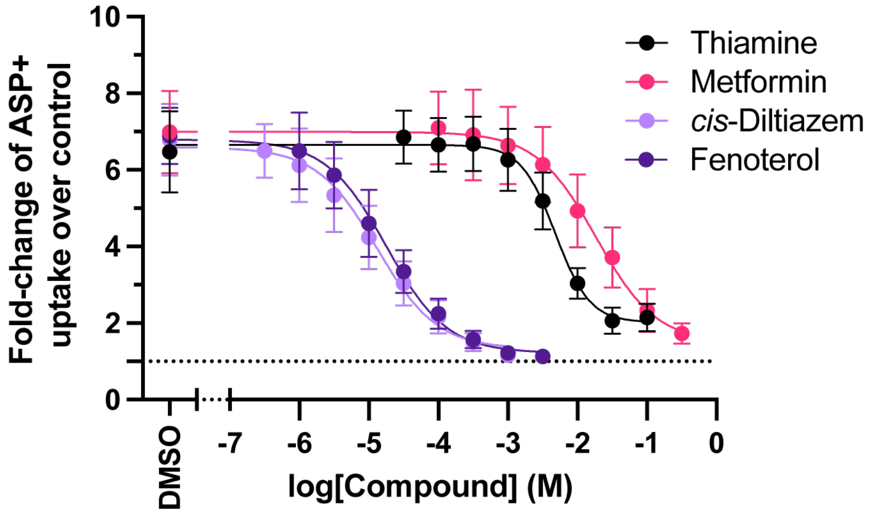

Supplementary Figure 1. Potency of compounds on the inhibition of uptake of the model OCT1-substrate ASP+. Inhibition of the uptake of the model OCT-substrate ASP+ of compounds presented in this study. OCT1-FLAG HEK293 cells were pre-incubated with compounds for 20 min prior to addition of 2 µM of the fluorescent substrate ASP^{+}. After 2 min, the measured fluorescence was normalized as a fold change over the negative control (doxycycline-uninduced OCT1-FLAG cells, dotted line) with n = 6 biological replicates for each compound, with errors shown as standard deviations.

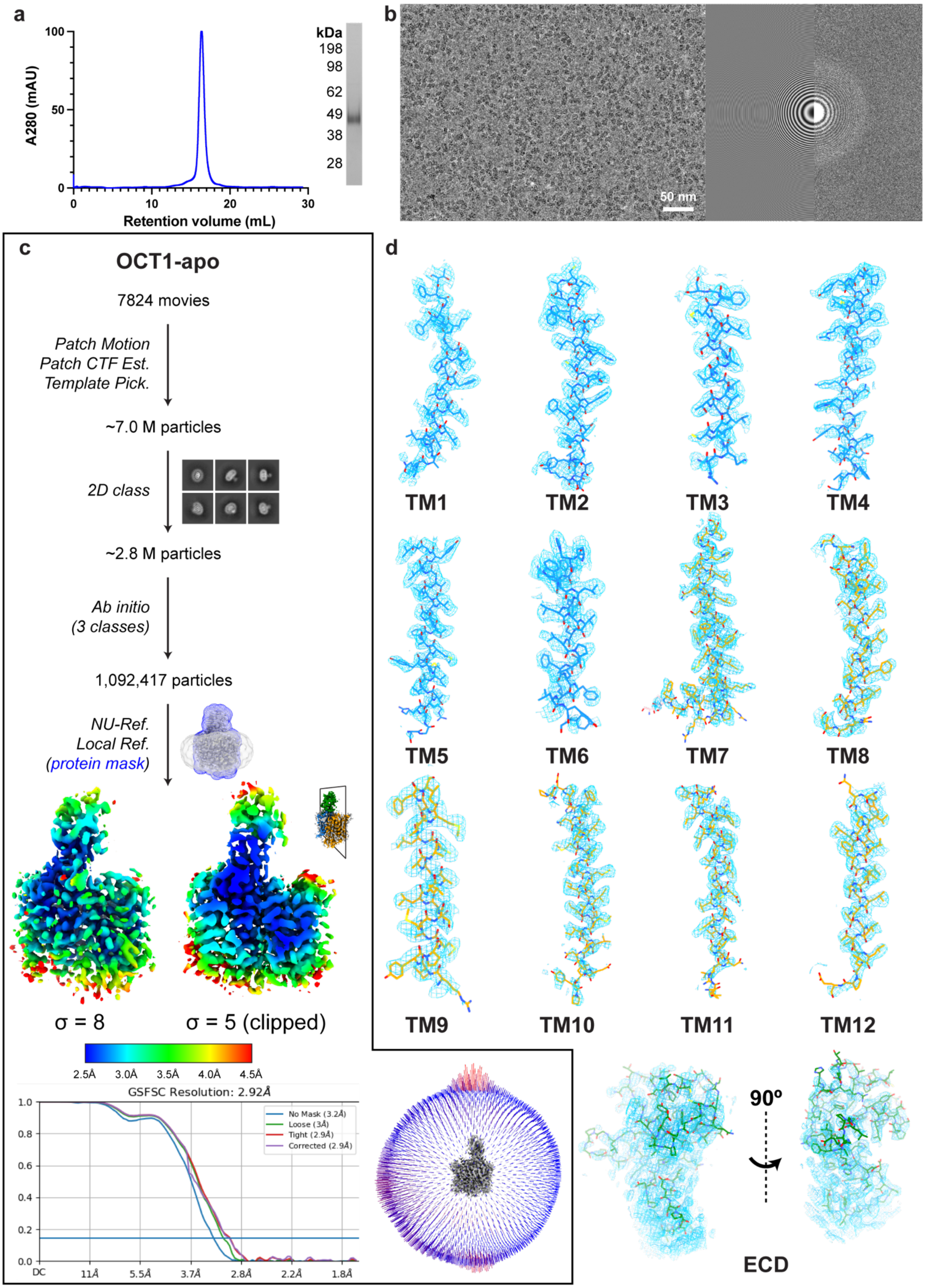

Supplementary Figure 2. Purification and Cryo-EM processing of OCT1-apo. (**a**) Size-exclusion chromatogram and SDS-PAGE gel of purified human OCT1. (**b**) Representative micrograph and corresponding CTF power spectra of OCT1-apo. (**c**) Cryo-EM processing workflow for OCT1-apo. From top to bottom: workflow, local resolution estimates, Fourier shell correlation curve, angular distribution of particles used in final reconstruction (**d**) Cryo-EM density of sharpened OCT1-apo map against model. The map was contoured between σ = 6-9.

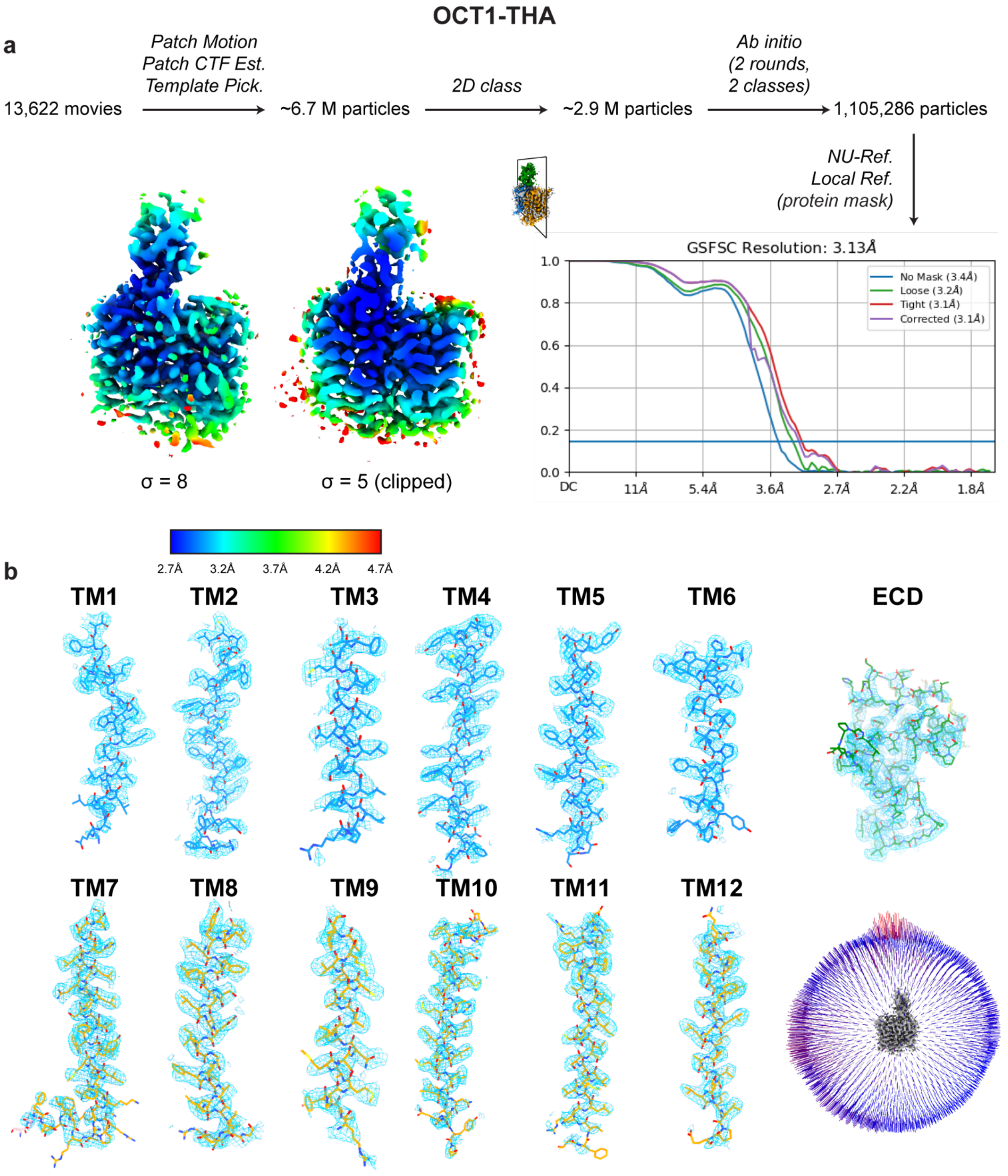

Supplementary Figure 3. Cryo-EM processing of OCT1-THA. (**a**) Cryo-EM processing workflow, local resolution estimates, Fourier shell correlation curves. (**b**) Cryo-EM density of sharpened map against model, contoured between σ = 6-9; and angular distribution of particles used in final reconstruction.

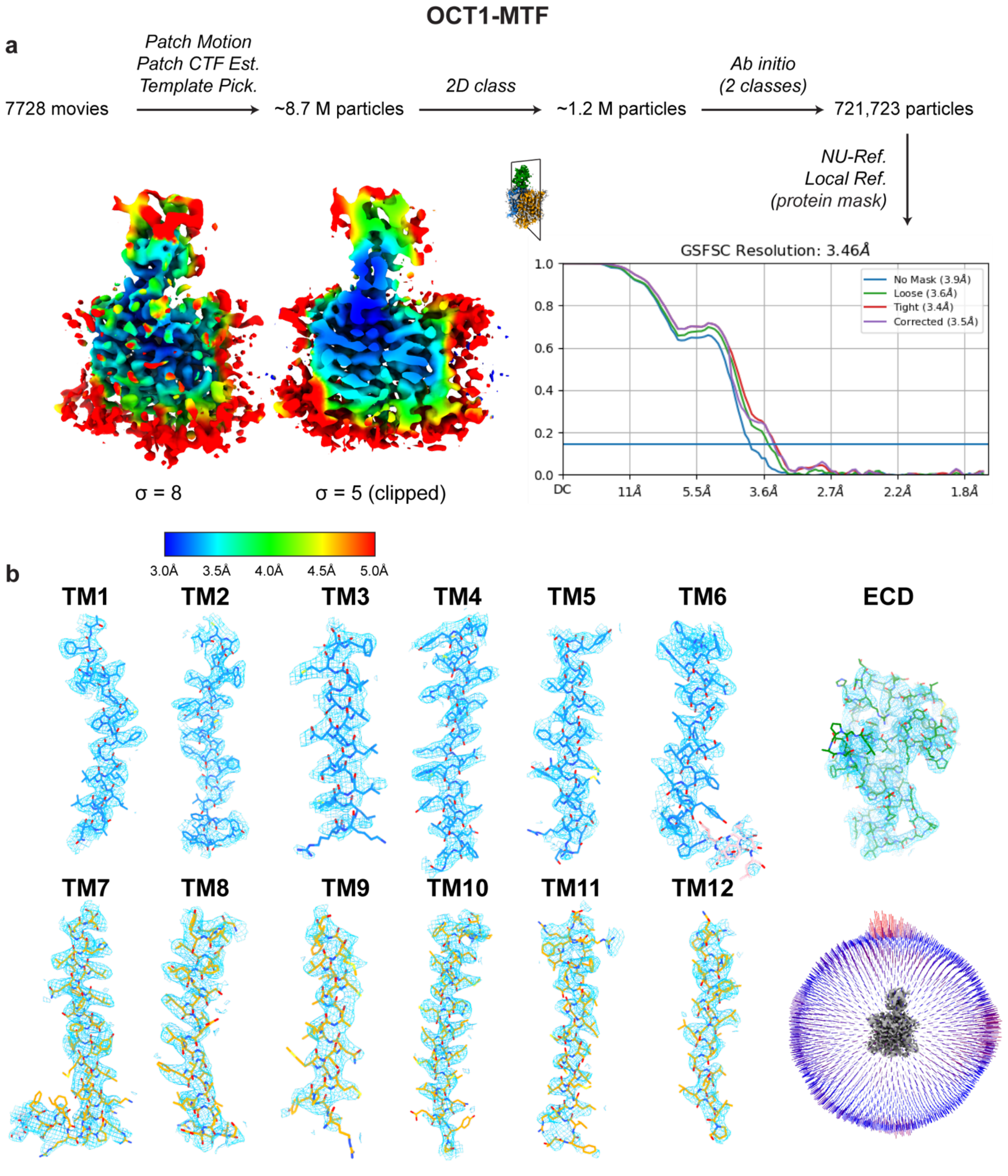

Supplementary Figure 4. Cryo-EM processing of OCT1-MTF. (**a**) Cryo-EM processing workflow, local resolution estimates, Fourier shell correlation curves. (**b**) Cryo-EM density of sharpened map against model, contoured between σ = 6-9; and angular distribution of particles used in final reconstruction.

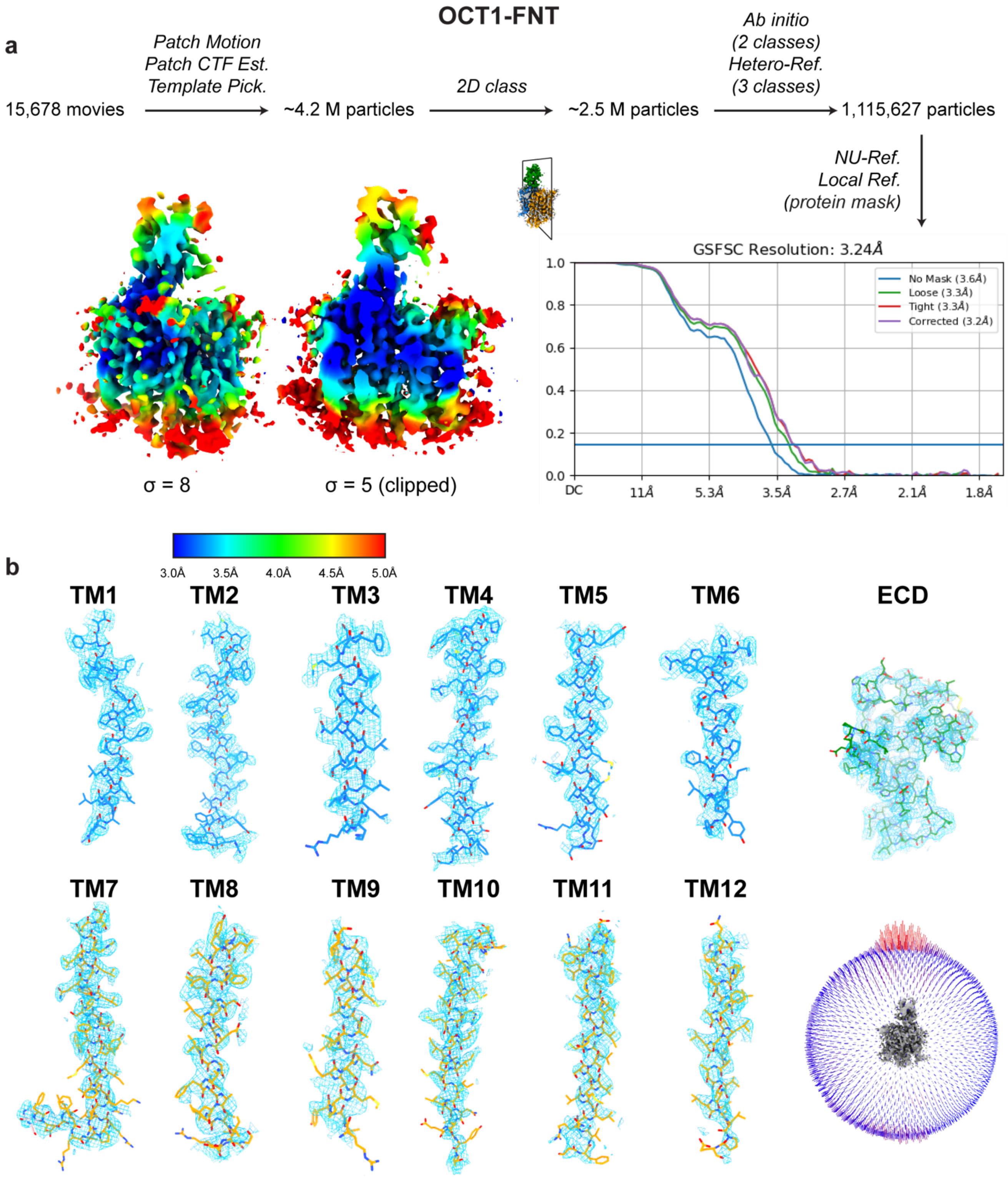

Supplementary Figure 5. Cryo-EM processing of OCT1-FNT. (**a**) Cryo-EM processing workflow, local resolution estimates, Fourier shell correlation curves. (**b**) Cryo-EM density of sharpened map against model, contoured between σ = 6-9; and angular distribution of particles used in final reconstruction.

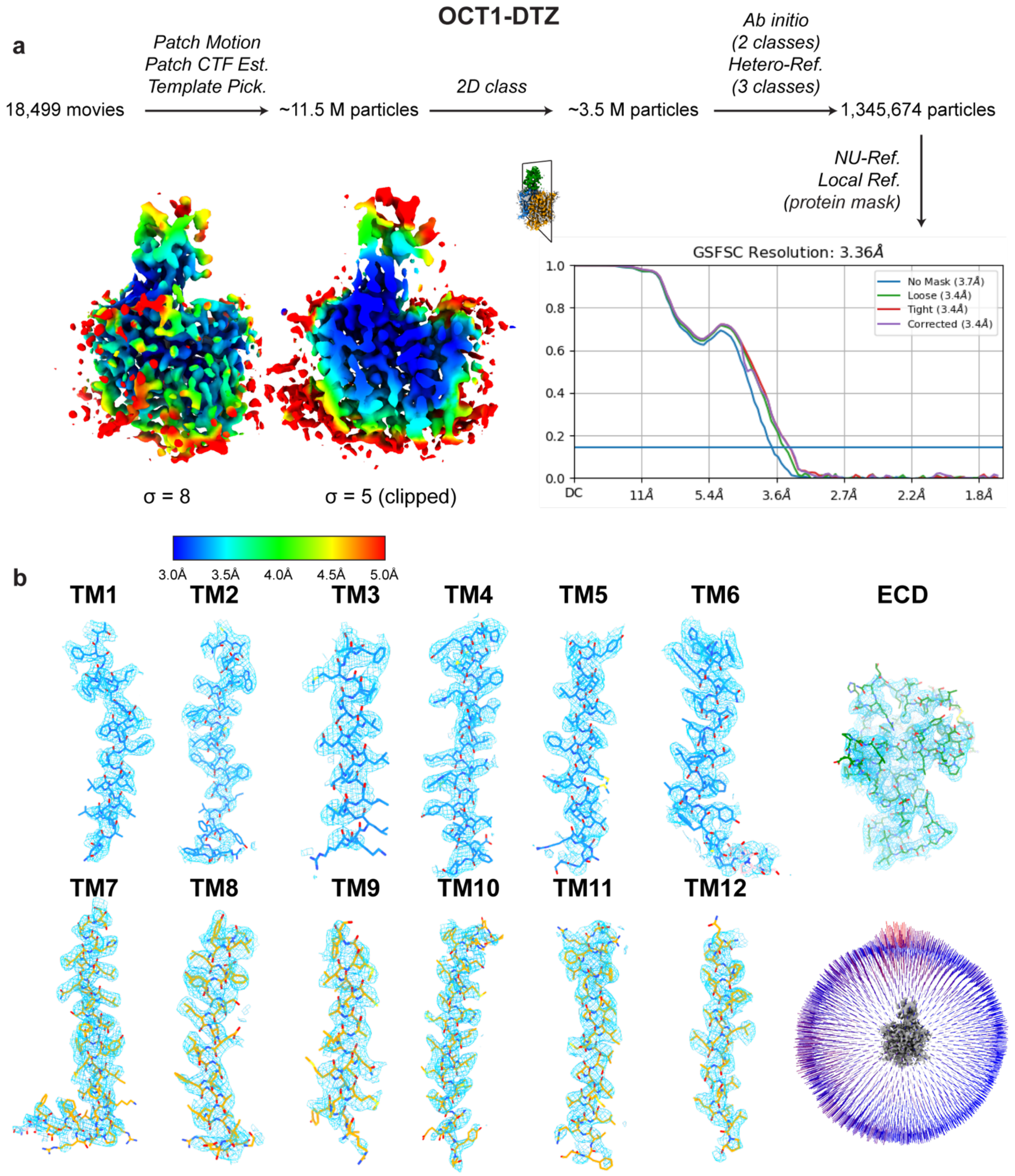

Supplementary Figure 6. Cryo-EM processing of OCT1-DTZ. (**a**) Cryo-EM processing workflow, local resolution estimates, Fourier shell correlation curves. (**b**) Cryo-EM density of sharpened map against model, contoured between σ = 6-9; and angular distribution of particles used in final reconstruction.

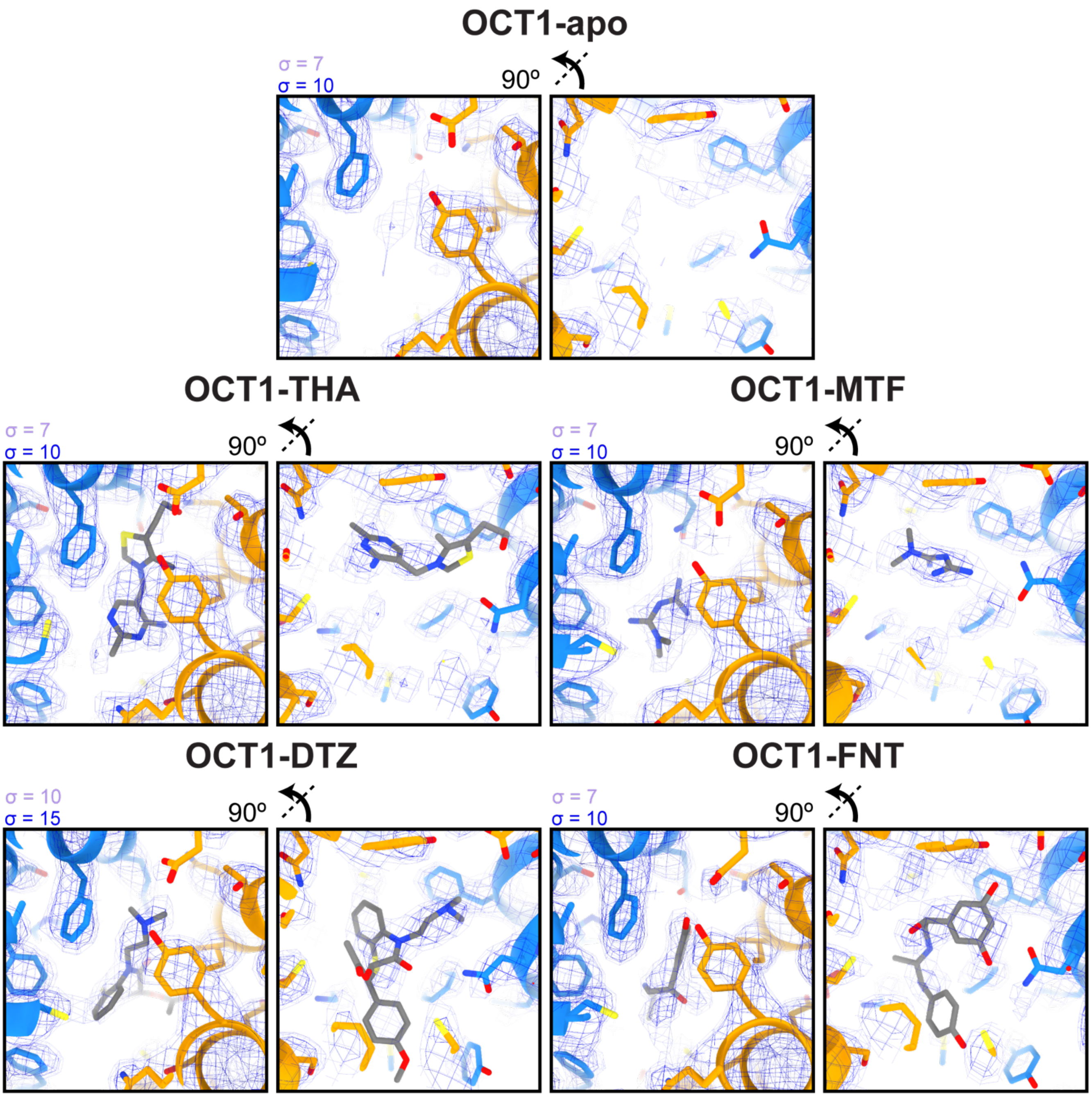

Supplementary Figure 7. Cryo-EM map density around substrate binding site. Close-up views of substrate binding site of modelled coordinates and density map (at two contour levels) for all cryo-EM maps in this study.

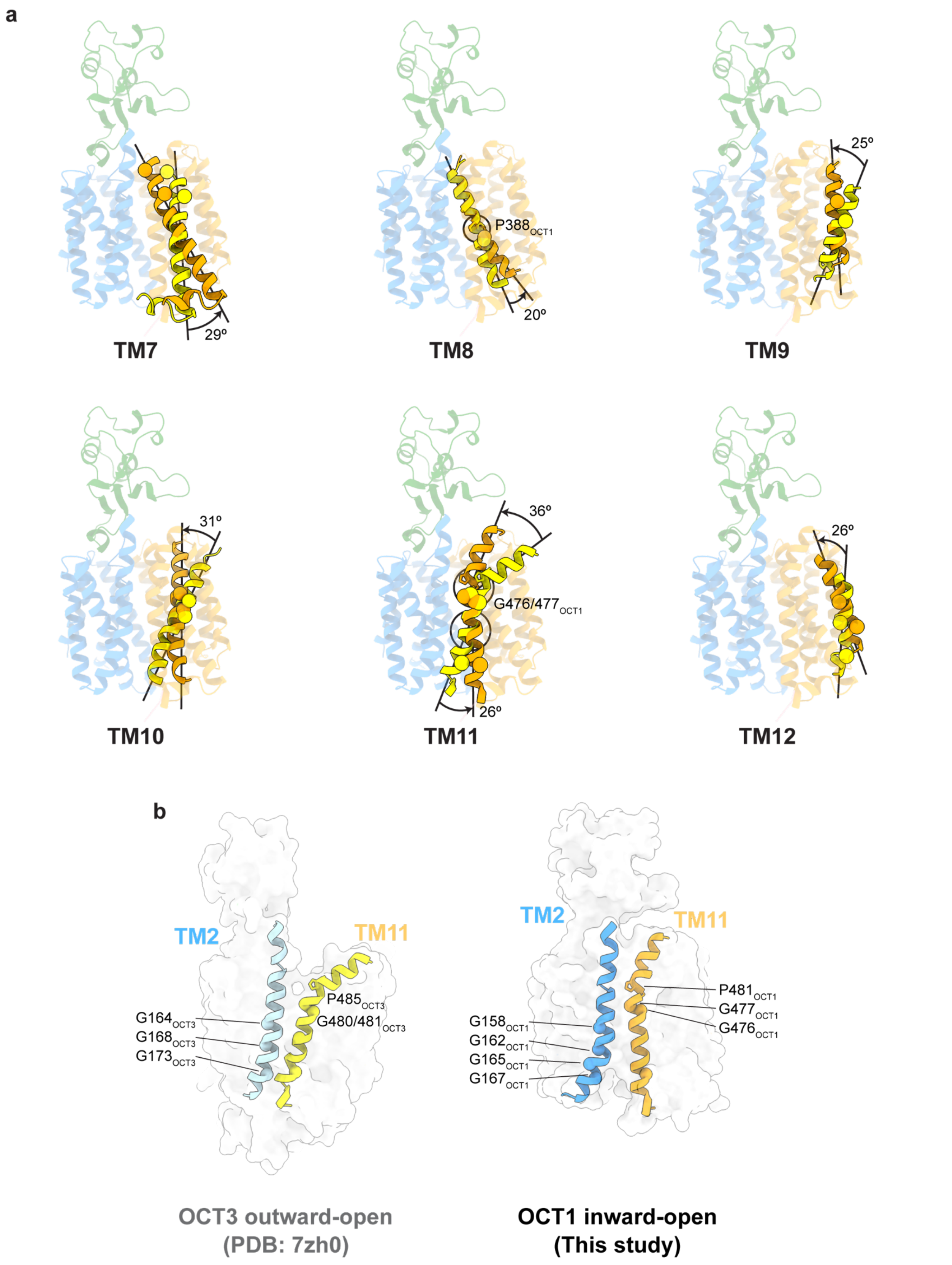

Supplementary Figure 8. Transmembrane rotations between inward-open OCT1 and outward-open OCT3. (**a**) Rotations between OCT1 inward-open and OCT3 outward-open (PDB: 7zh0)[1] for each TM. Key proline residues that hinge helix breaks are indicated and glycine residues along each TM are shown as spheres. Rotation was calculated using the Pymol Script Collection[2]. (**b**) Fenestration to the upper-leaflet between TM 2 and TM 11 in OCT3 outward-open hinges at $P485_{OCT3}$, which closes and in OCT1 inward-open with opening of the inner-leaflet fenestration through flexibility of TM 2 mediated by a series of glycine residues.

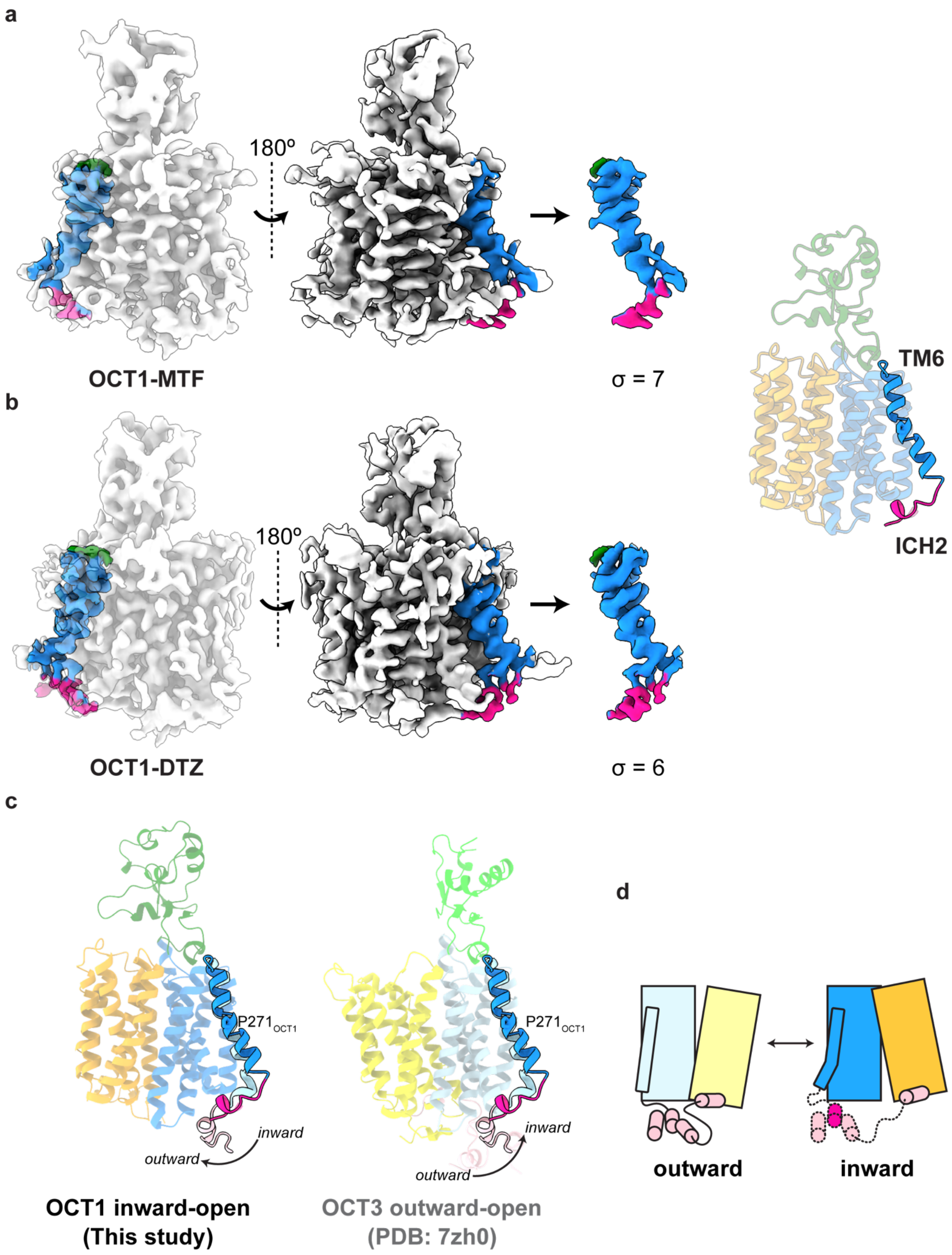

Supplementary Figure 9. Additional density in OCT1-MTF and OCT1-DTZ suggests TM 6 is flexible and moves laterally with ICH 2 in the inward-open state. (**a**) Map of OCT1-MTF (contoured to σ = 6) and (**b**) map of OCT1-DTZ (contoured to σ = 7) showing location of additional density corresponding to TM 6 (blue) and part of ICH 2 (magenta). (**c**) Comparison of modelled TM 6 and ICH 2 of OCT1 to OCT3, showing rotation from a helix break at $P271_{OCT1}$. (**d**) Schematic of movements of ICHs with TM 6 between outward- and inward-facing conformations.

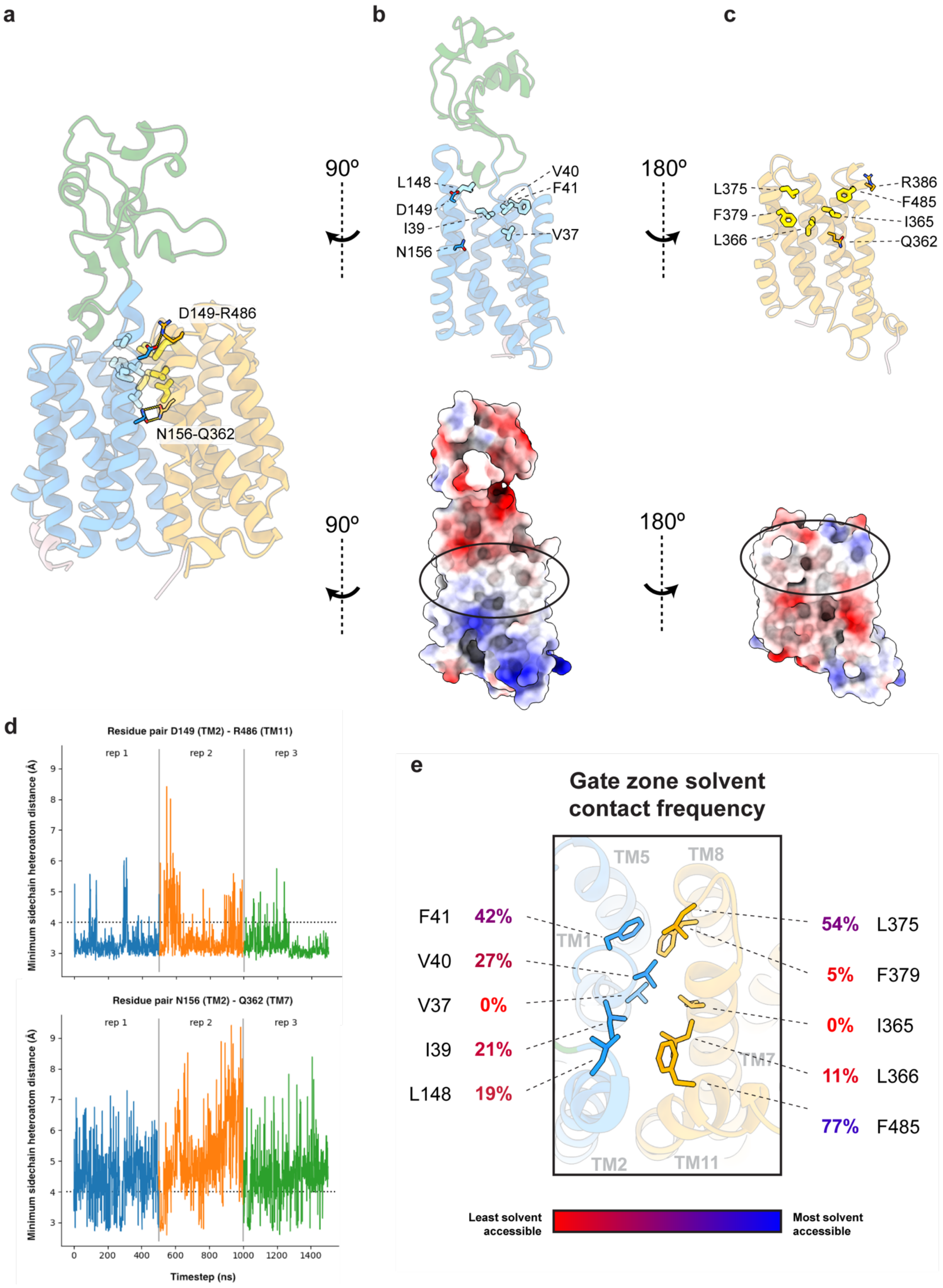

Supplementary Figure 10. Interactions at the extracellular gate of OCT1 are mediated through hydrophobic and polar interactions. (**a**) Overview of residues involved in extracellular gating, with polar extracellular gating pairs labelled. (**b**) View of N-terminal lobe face showing locations of polar and hydrophobic residues and the hydrophobic patch of the electrostatic potential surface. (**c**) View of C-terminal lobe face showing locations of polar and hydrophobic residues and the hydrophobic patch of the electrostatic potential surface. (**d**) Distance analysis of polar gating residues (D149-R486, top; N156-Q362, bottom) from

MD simulations. A reference line is drawn at 4 Å. The mean minimum distance between the sidechain heteroatoms of residue pair D149-R486 is 3.4 ± 0.6 Å. The mean minimum distance between the sidechain heteroatoms of residue pair N156-Q362 is 4.7 ± 1.1 Å. (**e**) The gate zone solvent contact frequency was defined as the percentage of simulation frames in which any residue atom is within 3 Å of any solvent atom where that atom was located between 65 and 70 Å in the Z-axis, after the protein had been aligned to the transmembrane domain backbone. This range was chosen to exclude contact with water molecules in the extracellular solvent, or in the substrate binding cavity. Total solvent contact frequency is also listed in **Supplementary Table 6**.

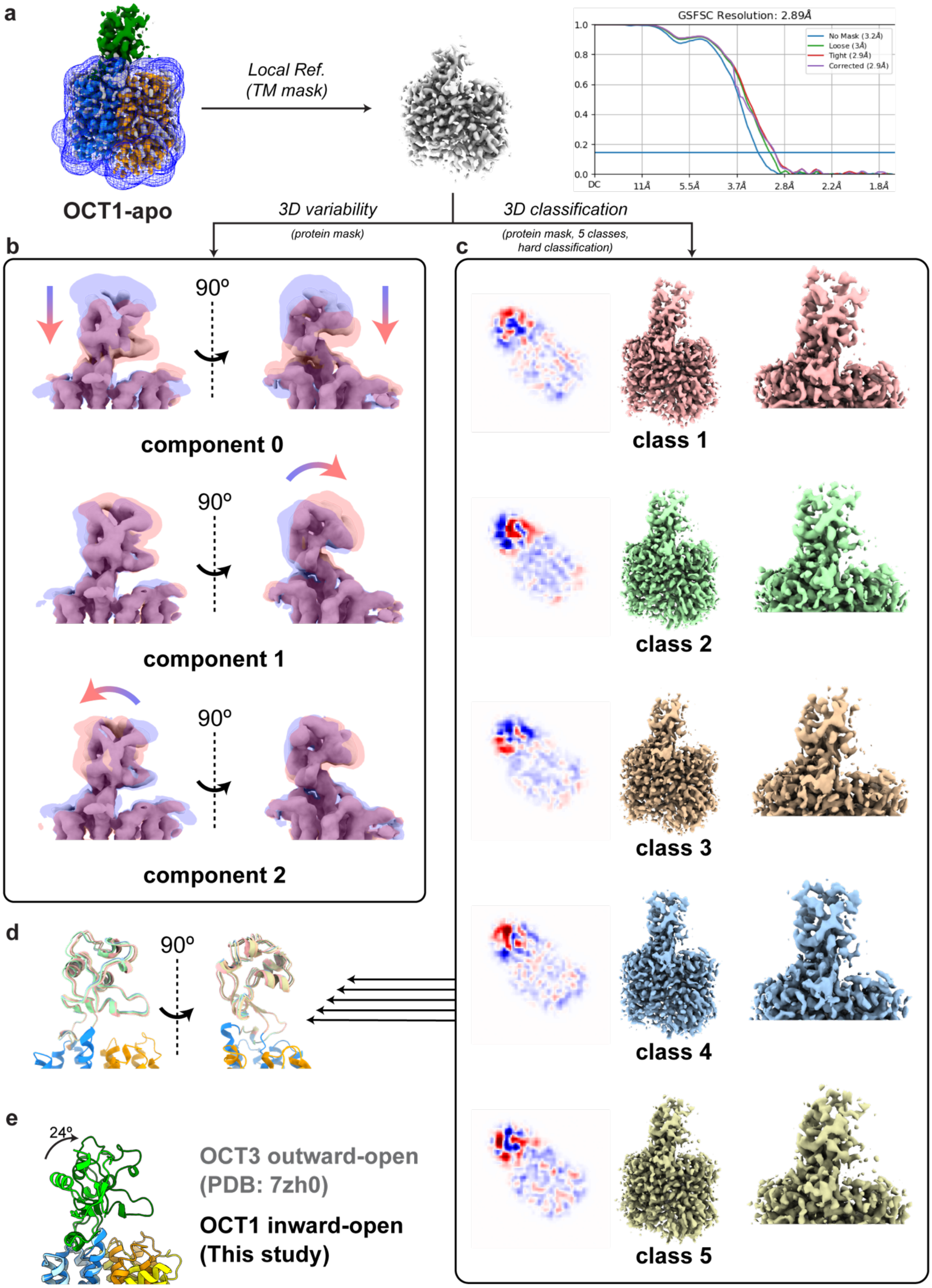

Supplementary Figure 11. Conformational stability of the ECD in OCT1. (**a**) Cryo-EM strategy for observing variability in the ECD. A soft mask (blue) of the transmembrane region was used for focused local refinement in cryoSPARC[3]. Aligned particles were then subject to 3D variability analysis or 3D classification. (**b**) Movements of the three components from 3D variability analysis, with 3% min/max percentile shown in the blue/red silhouette. Transitions from min to max indicated by the arrow. (**c**) 3D classification showing minimal movements of the ECD cryo-EM density when (**d**) the ECD was flexibly fitted in Coot[4]. (**e**) Relative rotation of the ECD of inward-open OCT1-apo to outward-open OCT3. Rotation was calculated using the Pymol Script Collection[2].

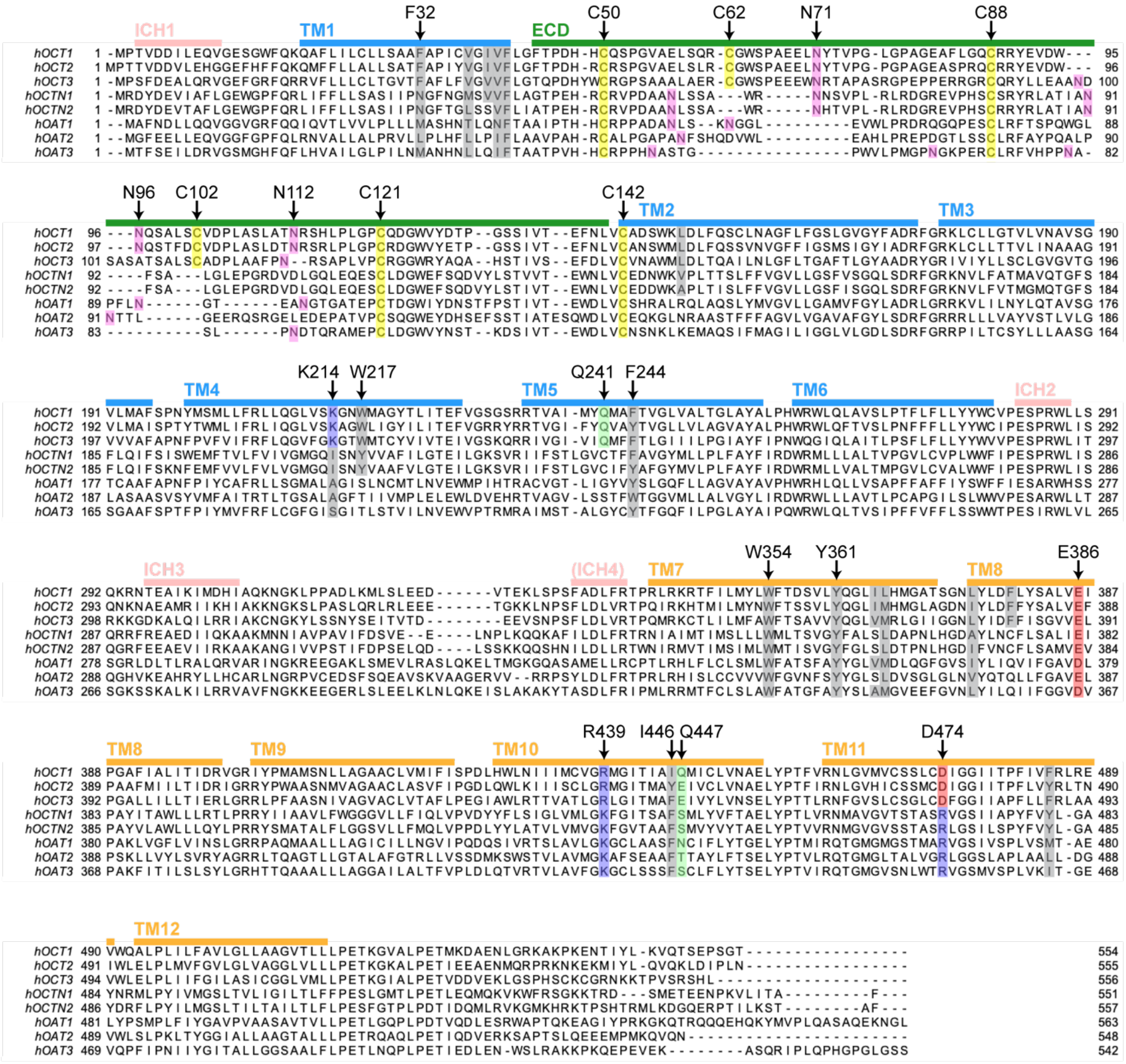

Supplementary Figure 12. Sequence alignment of SLC22A1-8. The sequences of human SLC22A1 to SLC22A8 (OCT1, OCT2, OCT3, OCTN1, OCTN2, OAT1, OAT2, OAT3, respectively) were aligned in Clustal Omega[5] and visualized in Jalview[6]. Key OCT1 residue positions outlined in this study are indicated.

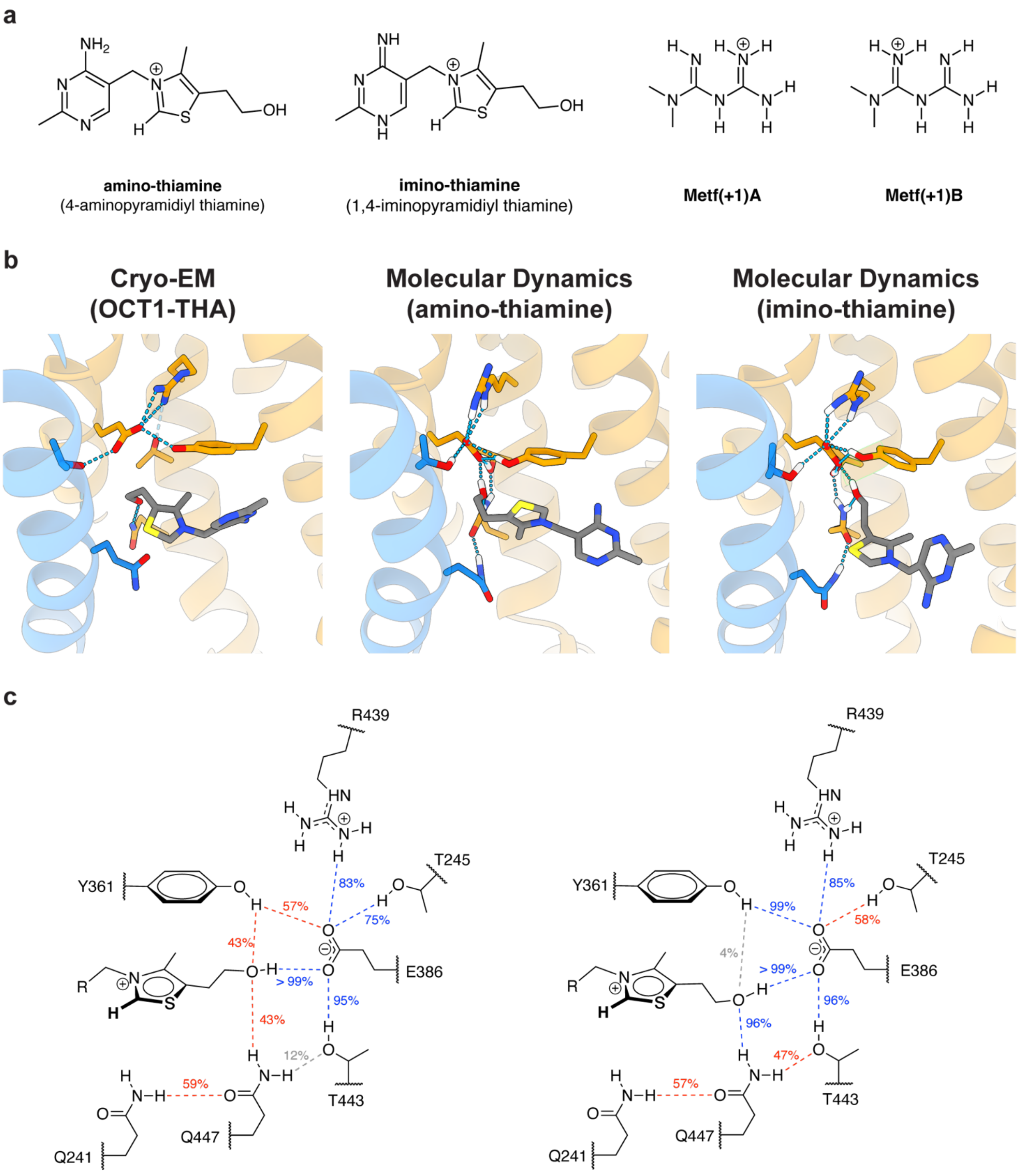

Supplementary Figure 13. Molecular dynamics simulations of low-affinity substrates of OCT1 reveal a hydrogen bonding network around E386. (**a**) Representation of tautomers of thiamine and metformin investigated in MD simulations. (**b**) Comparison of hydrogen-bonding networks in cryo-EM and MD simulations with both tautomers. (**c**) Hydrogen bonding interactions in MD simulations for amino-thiamine and imino-thiamine tautomers, 500 ns simulations performed in n = 3 replicates for each tautomer.

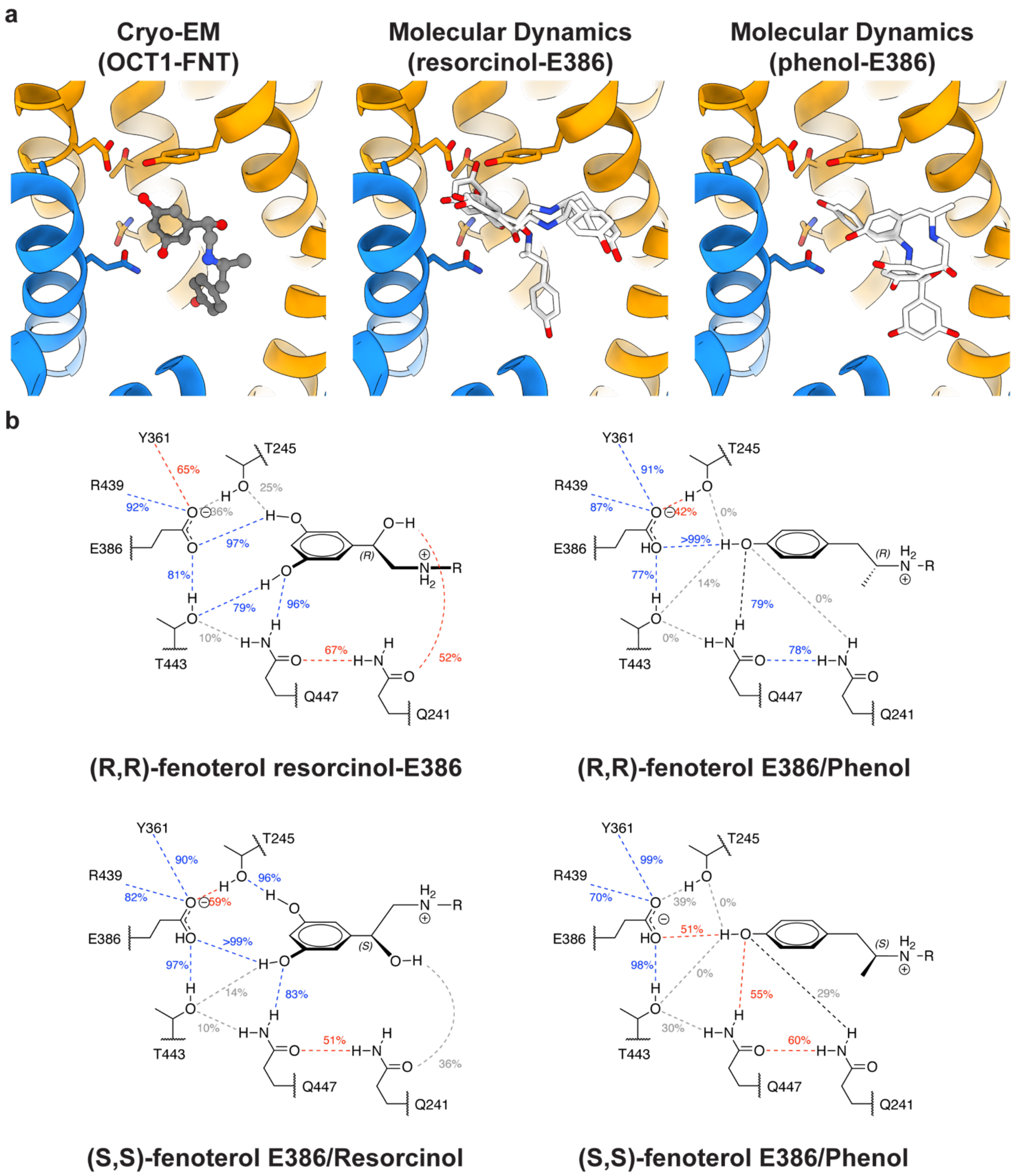

Supplementary Figure 14. Hydrogen-bonding network of fenoterol for different orientations of fenoterol. (**a**) Fenoterol observed in cryo-EM (OCT1-FNT), and representative conformations from MD simulations after initial docking with resorcinol or phenol groups positioned near E386. (**b**) Hydrogen bonding interactions in MD simulations for fenoterol stereoisomers with resorcinol or phenol positioned near E386, showing increased hydrogen bonding interactions in resorcinol-E386 orientation. 500 ns simulations performed in n = 3 replicates.

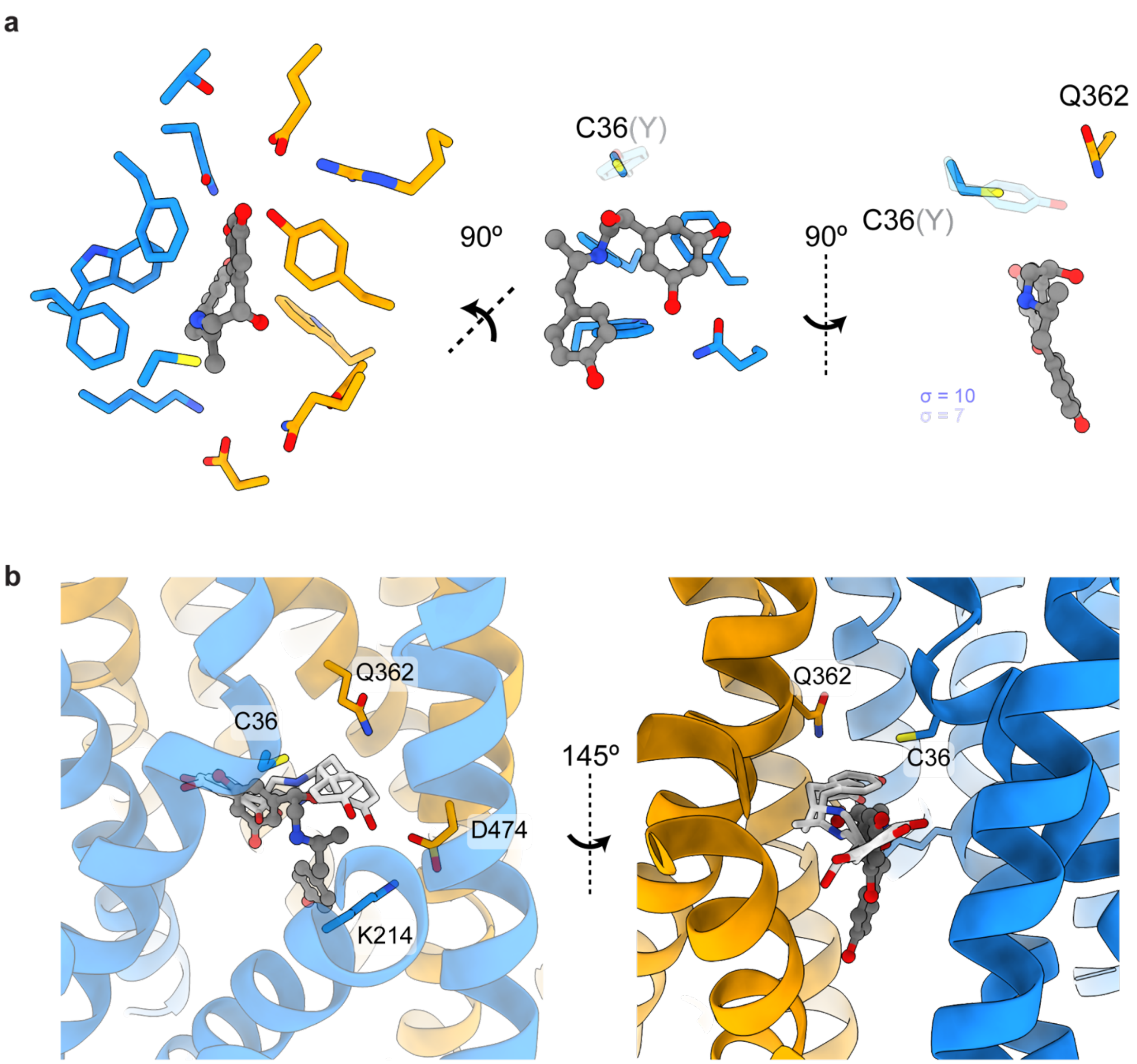

Supplementary Figure 15. Differences in fenoterol transport between human and mouse OCT1. (**a**) Cryo-EM model of fenoterol and positions of C36. The substitution C36Y as found in mouse OCT1 is shown in a light blue overlay. (**b**) Representative conformations sampled in MD simulations of fenoterol (both (R,R) and (S,S) isomers) with the phenol ring extended near C36.

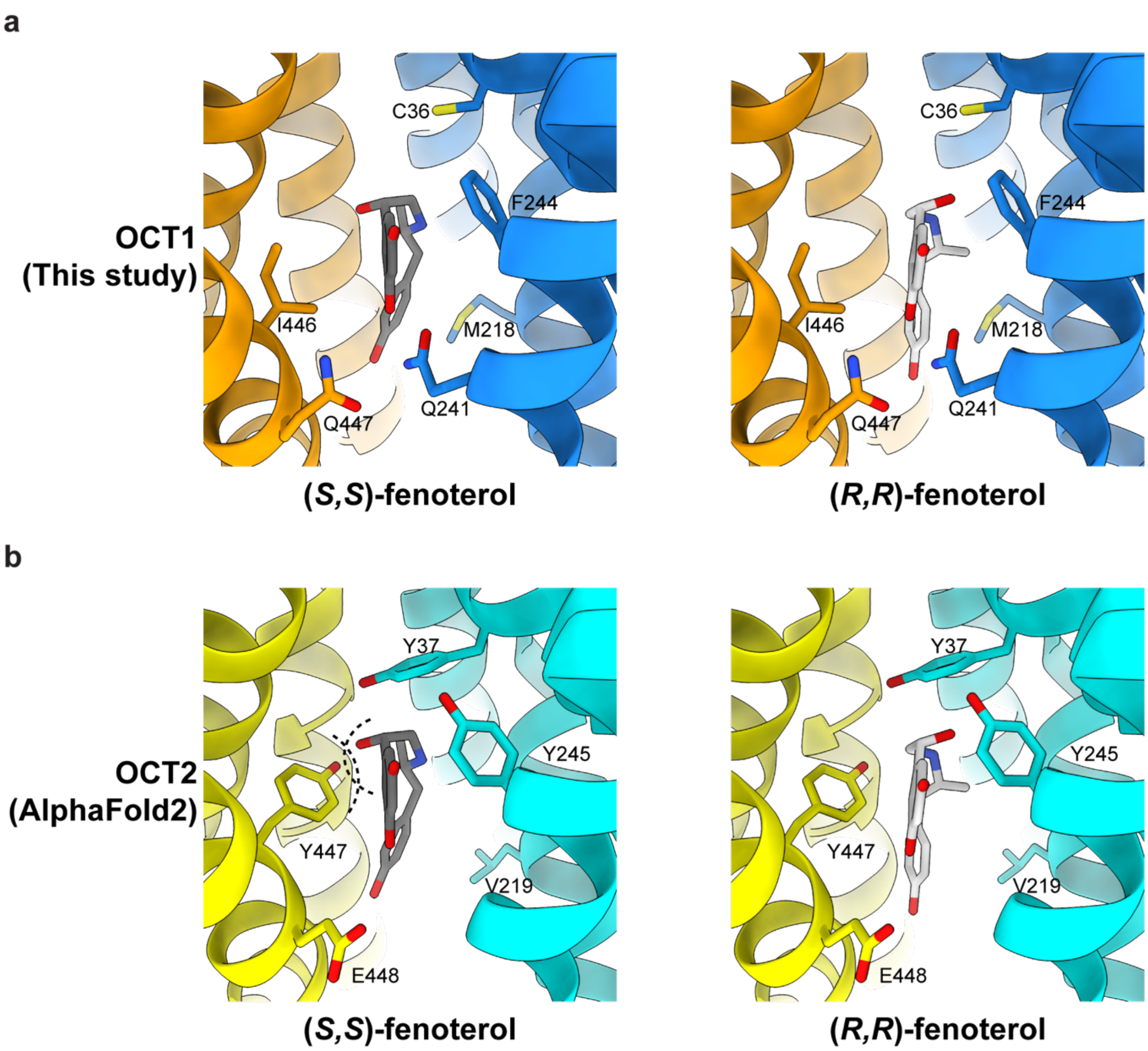

Supplementary Figure 16. Stereoselectivity to fenoterol in OCT1 and OCT2. (**a**) Comparison of modelled (*R,R*)- and (*S,S*)-fenoterol in the OCT1 binding pocket. (**b**) Comparison of modelled (*R,R*)- and (*S,S*)-fenoterol in the OCT2 binding pocket using the predicted inward-open structure of OCT2 generated by AlphaFold[7].

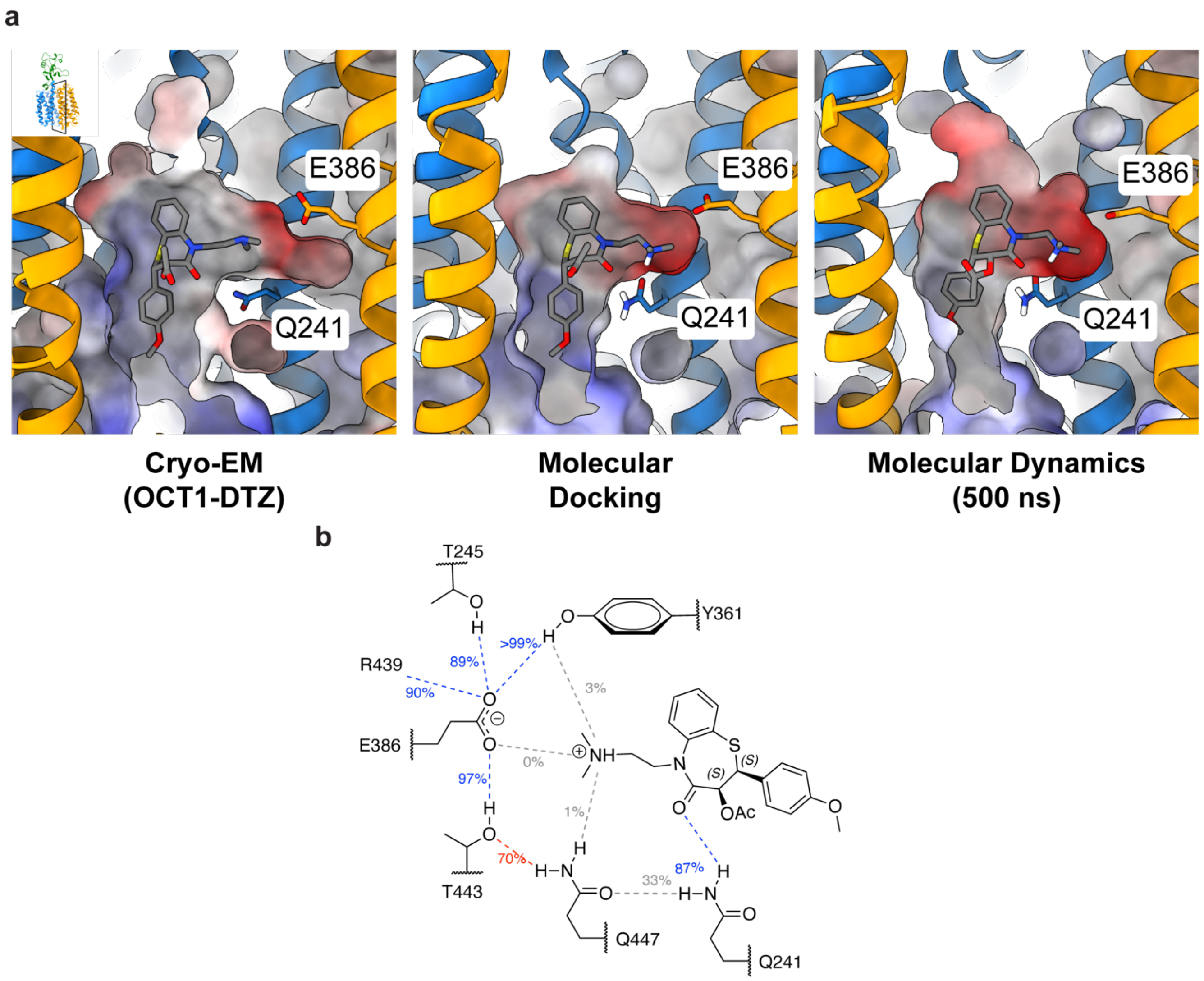

Supplementary Figure 17. Binding pocket of OCT1-DTZ. (**a**) Electrostatic surface potential (generated in ChimeraX) of OCT1 with diltiazem in cryo-EM (OCT1-DTZ), initially docked pose, and after 500 ns of MD simulation. (**b**) Hydrogen bonding interactions in MD simulations of diltiazem. Note that the ionic interaction of the pendant amine of diltiazem and E386 is not considered in this analysis.

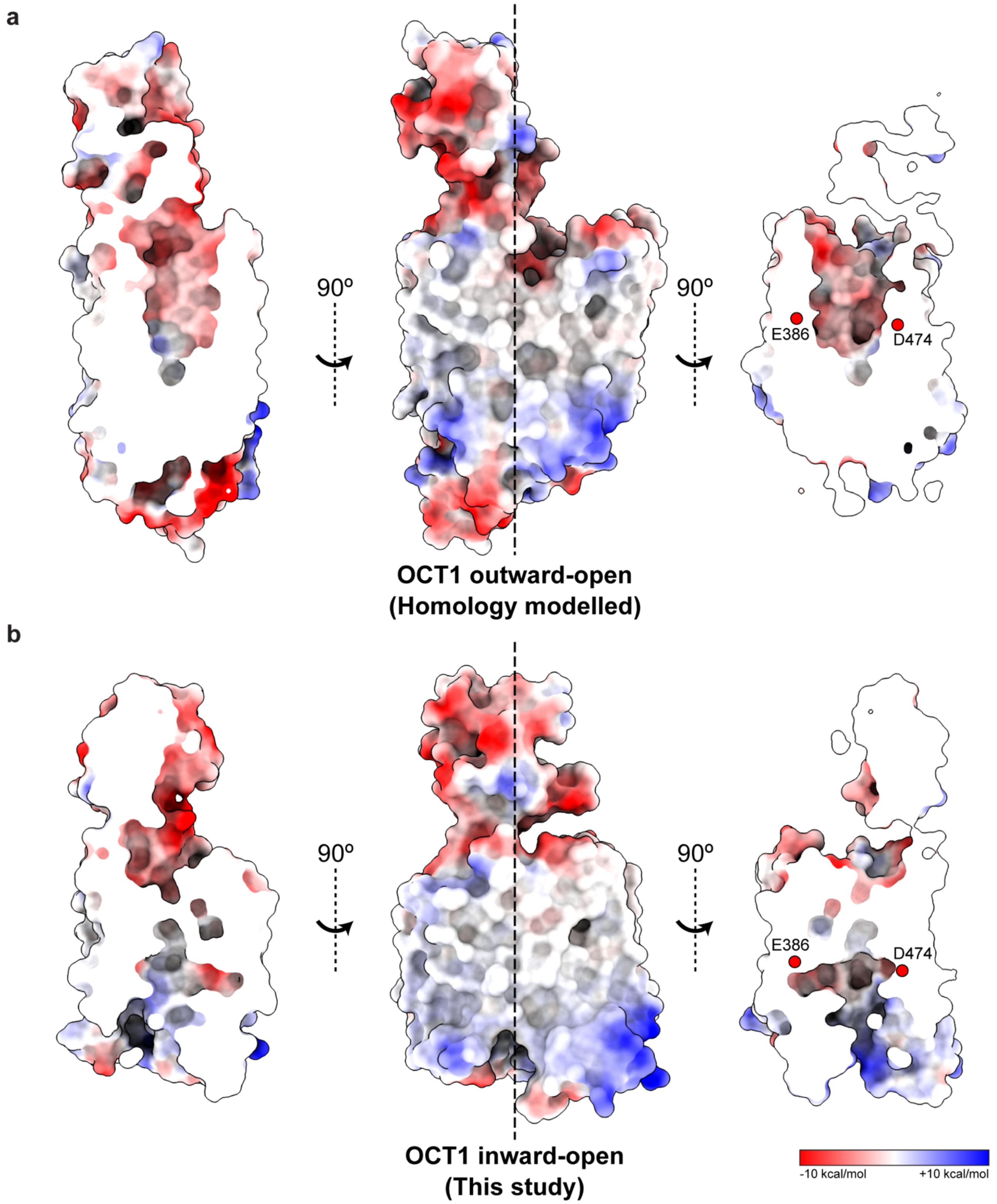

Supplementary Figure 18. Electrostatic potential of OCT1 in inward- and outward-facing conformations. (**a**) Electrostatic surface potential of an outward-facing OCT1 homology model using OCT3 (PDB: 7zh0)[1] as a template (generated using the Swiss-Model server[8]), with clipping of the N- (left) and C-terminal (right) domains to show the locations of E386/D474 and the binding site. (**b**) Electrostatic surface potential of OCT1-apo with clipping of the N- (left) and C-terminal (right) domains to show the locations of E386/D474 and the binding site. Coulombic electrostatic surface potential generated in ChimeraX[9].

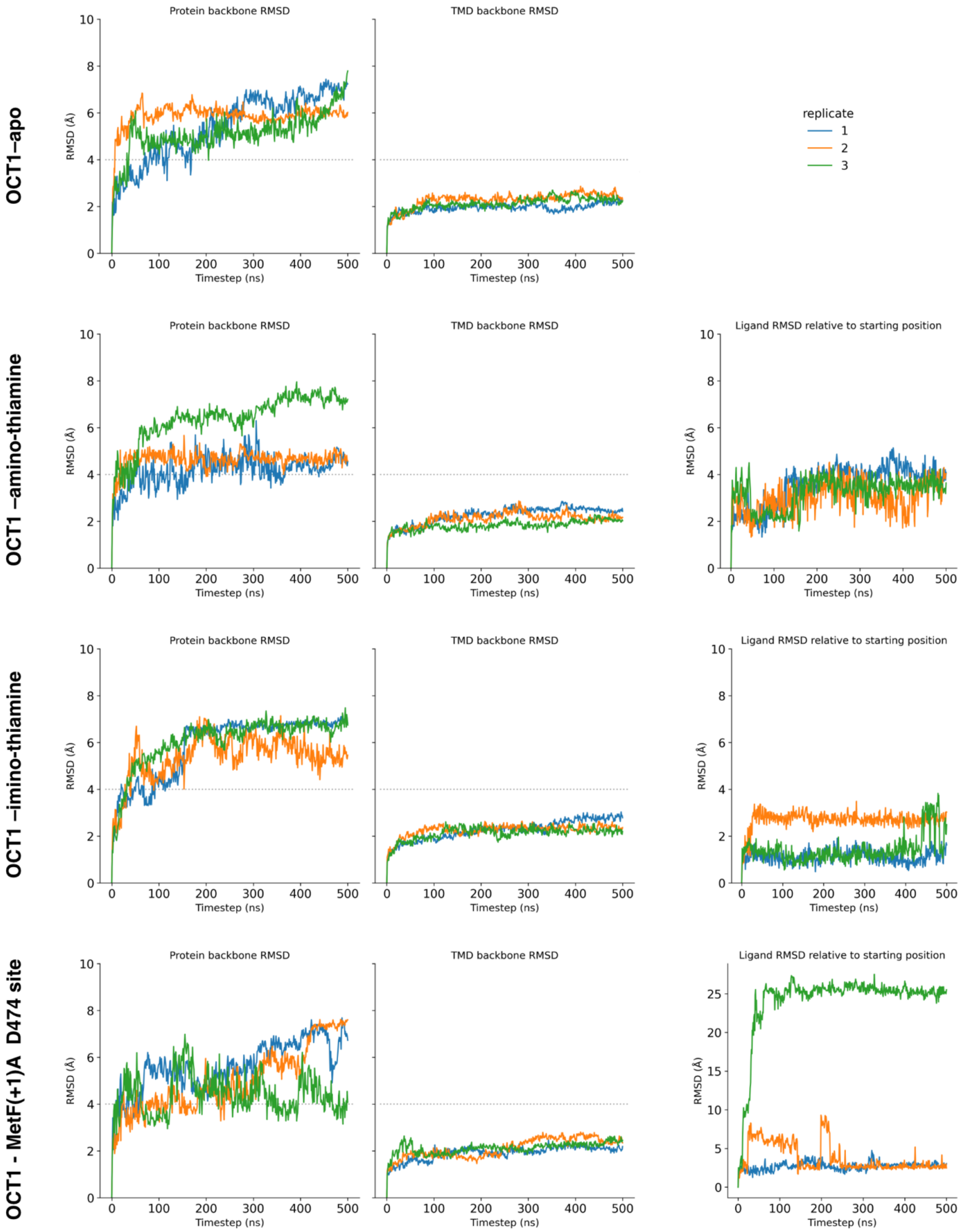

Supplementary Figure 19 (cont.)

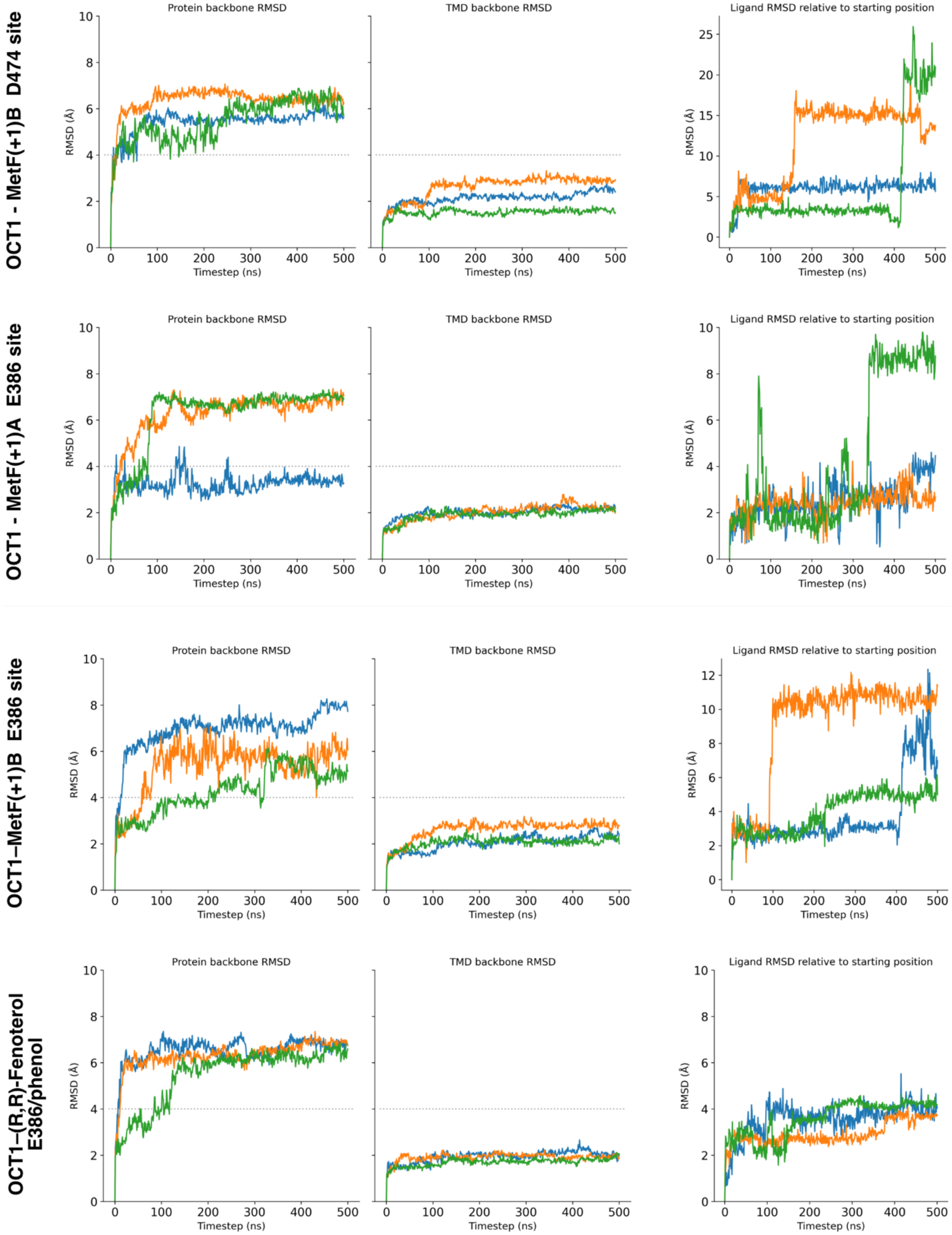

Supplementary Figure 19 (cont.)

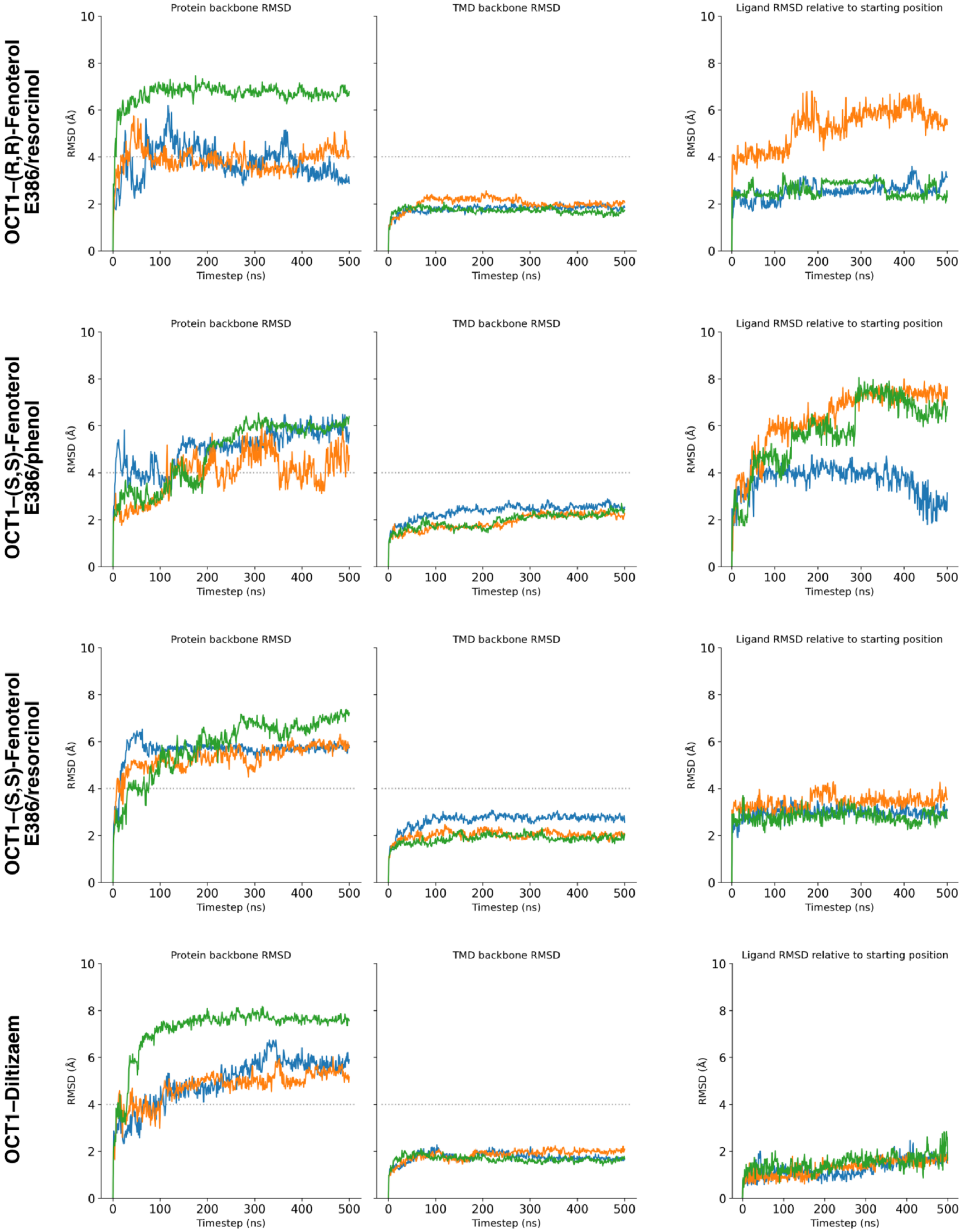

Supplementary Figure 19. Molecular dynamics simulation root mean square deviation. The RMSD of the protein backbone, transmembrane domain (TMD) backbone, and ligand across each replicate of 500 ns MD simulations.

Supplementary Table 1. Potency of compounds on the inhibition of uptake of the model OCT1-substrate ASP+

**Compound**	**logIC_{50} (M, ± SD)**	**IC_{50} (μM)**
Thiamine	-2.34 ± 0.14	4580
Metformin	-1.58 ± 0.39	26300
Fenoterol	-4.76 ± 0.11	17.5
cis-(±)-Diltiazem	-4.85 ± 0.16	14.0

Supplementary Table 2. Cryo-EM data collection, refinement, and validation statistics

	OCT1-apo (EMDB-40334) (PDB 8SC1)	OCT1-THA (EMDB-40339) (PDB 8SC6)	OCT1-MTF (EMDB-40337) (PDB 8SC4)	OCT1-FNT (EMDB-40336) (PDB 8SC3)	OCT1-DTZ (EMDB-40335) (PDB 8SC2)
**Data collection and processing**					
Magnification	135 000x	135 000x	81 000x	135 000x	135 000x
Voltage (kV)	300	300	300	300	300
Electron exposure (e–/Å^2)	88	86	50	81	79
Defocus range (μm)	-0.5 to -2.0	-0.5 to -2.0	-0.5 to -2.0	-0.5 to -2.0	-0.5 to -2.0
Pixel size (Å)	0.86	0.84	0.528	0.83	0.84
Symmetry imposed	C1	C1	C1	C1	C1
Initial particle images (no.)	6,977,371	6,329,303	8,732,848	6,560,371	9,228,053
Final particle images (no.)	1,398,918	1,105,286	721,723	849,183	1,345,674
Map resolution (Å)	2.92	3.13	3.46	3.24	3.36
FSC threshold	0.143	0.143	0.143	0.143	0.143
**Refinement**					
Initial model used (PDB code)	AlphaFold	OCT1 apo	OCT1 apo	OCT1 apo	OCT1 apo
Model resolution (Å)	3.14	3.36	3.66	3.44	3.56
FSC (model) threshold	0.5	0.5	0.5	0.5	0.5
Map sharpening *B* factor (Å^2)	101.2	147.3	157.8	142.3	155.5
Model composition					
Non-hydrogen atoms	3,447	3,479	3,544	3,463	3,534
Protein residues	446	447	456	445	453
Ligands	-	1	1	1	1
B factors (Å^2)					
Protein	59.39	65.82	80.81	72.99	79.05
Ligand	-	69.46	63.65	75.16	68.07
R.M.S. deviations					
Bond lengths (Å)	0.003	0.003	0.004	0.003	0.003
Bond angles (°)	0.679	0.502	0.684	0.628	0.551
Validation					
MolProbity score	0.93	1.12	1.23	0.99	1.18
Clashscore	1.15	3.28	2.80	2.15	3.93
Poor rotamers (%)	0.00	0.81	0.00	0.00	0.00
Ramachandran plot					
Favored (%)	97.51	99.10	97.12	98.41	98.66
Allowed (%)	2.49	0.90	2.88	1.59	1.34
Disallowed (%)	0.00	0.00	0.00	0.00	0.00

Supplementary Table 3. Molecular dynamics simulation system compositions.

	Simulation system	System composition by molecule type						
		OCT1	POPC	Cholesterol	Ligand	Water	Na^+	Cl^-
**1**	hOCT1 apo	1	786	200	0	63526	313	314
**2**	hOCT1 Amino-Thiamine	1	786	200	1	54961	285	287
**3**	hOCT1 Imino-Thiamine	1	786	200	1	54961	285	287
**4**	hOCT1 Metf(+1)A D474 site	1	786	200	1	54959	285	287
**5**	hOCT1 Metf(+1)A E386 site	1	786	200	1	55252	285	287
**6**	hOCT1 Metf(+1)B D474 site	1	786	200	1	54960	285	287
**7**	hOCT1 Metf(+1)B E386 site	1	786	200	1	55928	285	287
**8**	hOCT1_R,R-Fenoterol E386-phenol	1	786	200	1	54960	285	287
**9**	hOCT1_R,R-Fenoterol E386-resorcinol	1	786	200	1	55936	285	287
**10**	hOCT1_S,S-Fenoterol E386-phenol	1	786	200	1	55948	285	287
**11**	hOCT1_S,S-Fenoterol E386-resorcinol	1	786	200	1	58046	292	294
**12**	hOCT1 Diltiazem	1	786	200	1	55581	285	287

Supplementary Table 4. Protein backbone root mean squared deviations from molecular dynamics simulations.

	Simulation System	Protein backbone RMSD (Å)	TMD backbone RMSD (Å)	ECD backbone RMSD fit to ECD (Å)	ECD backbone RMSD fit to TMD (Å)
**1**	hOCT1 apo	5.5 ± 1.1	2.1 ± 0.3	5.1 ± 1.3	13.8 ± 3.1
**2**	hOCT1 Amino-Thiamine	4.3 ± 0.9	1.9 ± 0.2	4.6 ± 1.0	10.8 ± 2.6
**3**	hOCT1 Imino-Thiamine	5.1 ± 1.2	2.1 ± 0.3	5.3 ± 1.3	13.3 ± 4.1
**4**	hOCT1 Metf(+1)A D474 site	5.0 ± 1.2	2.1 ± 0.3	4.8 ± 1.1	12.7 ± 3.4
**5**	hOCT1 Metf(+1)A E386 site	5.2 ± 1.7	2.0 ± 0.3	4.8 ± 0.9	14.3 ± 6.1
**6**	hOCT1 Metf(+1)B D474 site	5.7 ± 0.8	2.1 ± 0.6	4.7 ± 1.0	15.6 ± 2.3
**7**	hOCT1 Metf(+1)B E386 site	5.5 ± 1.5	2.2 ± 0.4	4.9 ± 1.3	14.1 ± 4.4
**8**	hOCT1_(R,R)-Fenoterol E386-phenol	6.1 ± 1.0	1.9 ± 0.2	4.5 ± 0.6	17.0 ± 2.9
**9**	hOCT1_(R,R)-Fenoterol E386-resorcinol	4.8 ± 1.5	1.8 ± 0.2	4.8 ± 0.8	12.1 ± 5.3
**10**	hOCT1_(S,S)-Fenoterol E386-phenol	4.6 ± 1.2	2.1 ± 0.4	4.6 ± 1.0	11.3 ± 3.5
**11**	hOCT1_(S,S)-Fenoterol E386-resorcinol	5.6 ± 0.9	2.2 ± 0.4	4.9 ± 1.2	14.0 ± 2.9
**12**	hOCT1 Diltiazem	5.6 ± 1.5	1.8 ± 0.2	4.8 ± 0.9	15.3 ± 4.2

Supplementary Table 5. Ligand backbone root mean squared deviations for molecular dynamics simulations.

	Simulation System	Ligand RMSD relative to starting position (Å)			
		replicate 1	replicate 2	replicate 3	combined
**2**	hOCT1 Amino-Thiamine	3.6 ± 0.9	3.1 ± 0.7	3.2 ± 0.6	**3.3 ± 0.8**
**3**	hOCT1 Imino-Thiamine	1.1 ± 0.3	2.7 ± 0.4	1.4 ± 0.6	**1.7 ± 0.8**
**4**	hOCT1 MetF(+1)A D474 site	2.7 ± 0.5	3.8 ± 1.8	24.1 ± 4.5 [a]	**10.2 ± 10.2**
**5**	hOCT1 MetF(+1)A E386 site	2.5 ± 0.8	2.4 ± 0.5	4.3 ± 3.2 [b]	**3.1 ± 2.1**
**6**	hOCT1 MetF(+1)B D474 site	6.0 ± 1.1	11.9 ± 4.8 [a]	5.9 ± 6.2 [a]	**7.9 ± 5.3**
**7**	hOCT1 MetF(+1)B E386 site	3.8 ± 2.1 [b]	9.1 ± 3.1 [b]	4.0 ± 1.1	**5.6 ± 3.3**
**8**	hOCT1 (R,R)-Fenoterol E386-phenol	3.6 ± 0.7	2.9 ± 0.5	3.6 ± 0.8	**3.4 ± 0.7**
**9**	hOCT1 (R,R)-Fenoterol E386-resorcinol	2.5 ± 0.4	5.2 ± 0.9 [c]	2.6 ± 0.3	**3.5 ± 1.4**
**10**	hOCT1 (S,S)-Fenoterol E386-phenol	3.7 ± 0.6	6.3 ± 1.3 [d]	5.7 ± 1.5 [d]	**5.2 ± 1.7**
**11**	hOCT1 (S,S)-Fenoterol E386-resorcinol	3.0 ± 0.3	3.4 ± 0.3	2.8 ± 0.3	**3.1 ± 0.4**
**12**	hOCT1 Diltiazem	1.3 ± 0.4	1.3 ± 0.3	1.5 ± 0.3	**1.4 ± 0.4**

[a] Denotes replicates in which metformin was released from the OCT1 binding cavity

[b] Denotes replicates in which metformin diffused from the E386 proximal site to the D474 proximal site

[c] Denotes replicates in which (R,R)-fenoterol underwent a conformational change that positioned the phenol group adjacent to the intracellular gates

[d] Denotes replicates in which (S,S)-fenoterol diffused away from E386 to adopt a condensed conformation adjacent to D474

Supplementary Table 6. Solvent penetration into the apo-OCT1 hydrophobic gate from molecular dynamics simulations

Residue	Solvent contact frequency	Gate zone solvent contact frequency
**VAL37**	8.24%	0%
**ILE39**	36.24%	20.81%
**VAL40**	60.17%	28.66%
**PHE41**	90.23%	42.02%
**LEU148**	36.97%	18.68%
**ILE365**	24.53%	0%
**LEU366**	24.60%	11.04%
**LEU375**	59.97%	54.26%
**PHE379**	87.97%	4.72%
**PHE485**	97.75%	77.06%

The solvent contact frequency of an OCT1 residue is defined as the percentage of simulation frames in which any residue atom is within 3 Å of any solvent atom, including solvent atoms from the extracellular solvent, or the substrate binding cavity. A low solvent contact frequency indicates that solvent molecules rarely interact with these residues and are excluded from within the hydrophobic gate.

The gate zone solvent contact frequency was defined as the percentage of simulation frames in which any residue atom is within 3 Å of any solvent atom where that atom was located between 65 and 70 Å in the Z-axis, after the protein had been aligned to the transmembrane domain backbone. This range was chosen to exclude contact with water molecules in the extracellular solvent, or in the substrate binding cavity.

Supplementary Table 7. OCT1-thiamine contact frequencies from molecular dynamics simulations

Residue	Thiamine contact frequency		Thiazolium contact frequency	
	amino-thiamine	imino-thiamine	amino-thiamine	imino-thiamine
**PHE32**	47.9%	91.6%	32.9%	43.4%
**CYS36**	32.7%	86.4%	0.1%	7.7%
**PHE159**	22.7%	90.8%	n.c.	n.c.
**GLN241**	97.8%	94.8%	94.4%	77.1%
**MET242**	71.5%	85.2%	3.1%	0.1%
**PHE244**	95.9%	99.8%	86.8%	97.8%
**THR245**	86%	71.1%	1.4%	3.2%
**TYR361**	100%	100%	93%	84.7%
**GLN362**	53%	96.7%	2.1%	0.8%
**GLU386**	100%	99.6%	3.7%	0.1%
**THR443**	93.6%	97.5%	15.6%	0.5%
**GLN447**	91.5%	99.9%	46.9%	18.2%
**ASP474**	2.9%	84%	n.c.	n.c.

The contact frequency between any OCT1 residue and thiamine is defined as the percentage of simulation frames in which any residue atom is within 4 Å of any thiamine atom. The contact frequency between any one residue and the thiazolium ring is defined as the percentage of simulation frames in which any residue atom is within 4 Å of any atom in the thiazolium ring. n.c. indicates contact within 4 Å was not observed.

Supplementary Table 8. OCT1-metformin contact frequencies from molecular dynamics simulations

Residue	Metformin contact frequency			
	D474 site		E386 site	
	Metf(+1)A	Metf(+1)B	Metf(+1)A	Metf(+1)B
LYS214	51.33%	30.25%	9.97%	12.9%
TRP217	24.07%	30.72%	44.81%	14.43%
GLN241	35.04%	39.3%	87.17%	69.41%
MET242	n.c.	15.43%	81.72%	31.72%
PHE244	56.25%	57.58%	95.08%	64.76%
THR245	0.27%	1.99%	66.29%	39.89%
TYR361	57.65%	59.51%	98.34%	76.93%
GLN362	50.47%	36.1%	15.16%	33.38%
GLU386	0.27%	32.78%	83.44%	66.56%
THR443	0.27%	3.12%	77.73%	60.7%
ILE446	n.c.	30.98%	72.47%	41.16%
GLN447	23.4%	33.84%	85.44%	66.56%
ASP474	3.79%	35.84%	10.17%	32.91%

The contact frequency between any OCT1 residue and metformin is defined as the percentage of simulation frames in which any residue atom is within 4 Å of any metformin atom. n.c. indicates contact within 4 Å was not observed.

Supplementary Table 9. OCT1-fenoterol contact frequencies from molecular dynamics simulations of fenoterol docked with the resorcinol ring proximal to E386.

Residue	Fenoterol contact frequency		Resorcinol contact frequency		Phenol contact frequency		Ammonium contact frequency		Chiral hydroxyl contact frequency	
	FNRR	FNSS	FNRR	FNSS	FNRR	FNSS	FNRR	FNSS	FNRR	FNSS
**PHE32**	65.29%	54.45%	0.07%	n.c.	59.04%	53.72%	11.77%	0.20%	4.85%	0.07%
**CYS36**	59.71%	32.65%	n.c.	0.07%	56.25%	31.32%	16.76%	0.07%	0.47%	0.20%
**ASN156**	32.38%	28.32%	n.c.	n.c.	32.38%	28.32%	n.c.	n.c.	n.c.	n.c.
**PHE159**	63.43%	61.44%	n.c.	n.c.	63.43%	61.44%	n.c.	n.c.	n.c.	n.c.
**SER163**	29.12%	49.87%	n.c.	n.c.	29.12%	49.87%	n.c.	n.c.	n.c.	n.c.
**LYS214**	74.07%	99.60%	n.c.	n.c.	73.80%	99.60%	0.13%	0.20%	n.c.	n.c.
**TRP217**	79.79%	90.09%	7.85%	52.46%	36.77%	17.09%	8.38%	14.16%	49.00%	54.79%
**GLN241**	99.14%	99.93%	97.81%	99.80%	32.58%	1.53%	11.17%	28.72%	90.16%	61.57%
**MET242**	95.55%	74.27%	95.08%	74.27%	n.c.	n.c.	n.c.	n.c.	1.73%	0.40%
**PHE244**	98.74%	99.87%	98.27%	99.80%	18.75%	22.21%	13.30%	7.98%	22.07%	3.72%
**THR245**	72.74%	99.87%	72.74%	99.87%	n.c.	n.c.	n.c.	n.c.	n.c.	n.c.
**TRP354**	40.23%	43.62%	0.53%	n.c.	33.64%	17.22%	5.32%	17.22%	2.26%	1.40%
**SER358**	43.42%	58.38%	n.c.	n.c.	29.39%	42.82%	2.06%	0.20%	n.c.	n.c.
**TYR361**	100.00%	100.00%	99.93%	100.00%	1.80%	9.91%	31.32%	10.51%	0.27%	9.11%
**GLN362**	68.35%	72.81%	n.c.	0.07%	42.75%	63.96%	6.85%	3.52%	n.c.	0.07%
**GLU386**	99.47%	100%	99.47%	100%	n.c.	n.c.	n.c.	n.c.	n.c.	5.72%
**ARG439**	83.71%	58.05%	83.71%	58.05%	n.c.	n.c.	n.c.	n.c.	n.c.	n.c.
**THR443**	96.94%	98.74%	96.94%	98.67%	n.c.	n.c.	n.c.	n.c.	n.c.	23.14%
**ILE446**	93.55%	90.89%	89.89%	47.34%	5.59%	0.27%	17.49%	15.96%	19.81%	34.77%
**GLN447**	99.60%	96.48%	99.27%	95.41%	0.60%	n.c.	n.c.	9.11%	57.51%	39.83%
**CYS450**	37.90%	13.10%	3.86%	2.66%	30.52%	0.27%	16.29%	1.46%	20.94%	6.05%
**SER470**	41.49%	21.14%	n.c.	n.c.	41.49%	21.14%	n.c.	n.c.	n.c.	n.c.
**CYS473**	79.52%	95.61%	n.c.	n.c.	72.74%	94.81%	8.38%	10.64%	n.c.	0.07%
**ASP474**	66.49%	100%	n.c.	n.c.	66.49%	100%	n.c.	n.c.	n.c.	n.c.
**GLY477**	33.84%	41.95%	n.c.	n.c.	33.84%	41.95%	n.c.	n.c.	n.c.	n.c.

The contact frequency between any OCT1 residue and fenoterol is defined as the percentage of simulation frames in which any residue atom is within 4 Å of any fenoterol atom. The contact frequency between any OCT1 residue and the fenoterol resorcinol group is defined as the percentage of simulation frames in which any residue atom is within 4 Å of any atom in the fenoterol resorcinol ring or the resorcinol hydroxyl substituents. The contact frequency between any OCT1 residue and the fenoterol phenol group is defined as the percentage of simulation frames in which any residue atom is within 4 Å of any atom in the fenoterol phenol ring phenol hydroxyl group. The contact frequency between any OCT1 residue and the fenoterol ammonium is defined as the percentage of simulation frames in which any residue atom is within 4 Å of the nitrogen or hydrogen atoms of the fenoterol ammonium group. The contact frequency between any OCT1 residue and the fenoterol chiral hydroxyl is defined as the percentage of simulation frames in which any residue atom is within 4 Å of oxygen or hydrogen atoms of the fenoterol chiral hydroxyl group. n.c. indicates contact within 4 Å was not observed.

Supplementary Table 10. OCT1-diltiazem contact frequencies from molecular dynamics simulations

Residue	Diltiazem contact frequency	Diltiazem methoxyphenyl contact frequency	Diltiazem pendant ammonium contact frequency
**PHE32**	92.82%	0.53%	1.13%
**CYS36**	97.74%	n.c.	n.c.
**LYS214**	92.42%	54.52%	n.c.
**TRP217**	100%	99.73%	13.23%
**TYR221**	92.69%	92.69%	n.c.
**GLN241**	99.93%	97.87%	97.87%
**PHE244**	100%	0.53%	78.06%
**TRP354**	100%	100%	n.c.
**SER358**	88.16%	0.4%	n.c.
**TYR361**	99.8%	n.c.	90.82%
**GLN362**	90.76%	0.2%	n.c.
**GLU386**	65.89%	n.c.	65.89%
**ILE446**	99.53%	0.13%	56.72%
**GLN447**	94.95%	0.27%	94.88%
**SER470**	98.27%	98.27%	n.c.
**CYS473**	99.87%	69.95%	n.c.
**ASP474**	41.09%	3.39%	n.c.

The contact frequency between any OCT1 residue and diltiazem is defined as the percentage of simulation frames in which any residue atom is within 4 Å of any diltiazem atom. The contact frequency between any OCT1 residue and the diltiazem methoxyphenyl ring is defined as the percentage of simulation frames in which any residue atom is within 4 Å of any atom in the methoxyphenyl ring. The contact frequency between any OCT1 residue and the diltiazem pendant ammonium ring is defined as the percentage of simulation frames in which any residue atom is within 4 Å of any atom in the diltiazem dimethylammonium group. (n.c.) indicates contact within 4 Å was not observed. Contact with the cationic residue E386 is elevated but does not meet the threshold of frequency.

Supplementary Text 1. Parameterization details for thiamine and metformin tautomers.

At physiological pH, metformin can exist in multiple tautomerization states, with protonation of either the desmethylated guanidinium (MetF(+1)A) or the dimethylated guanidinium (MetF(+1)B) (Supplementary Fig. 13a). The two states are similar in energy (reported $\Delta E = 0.22$ kcal mol^{-1}), and both likely exist in an aqueous environment[10]. As such, molecular dynamics simulations were conducted with both tautomers.

The aminopyrimidal group of thiamine diphosphate has been shown to exist as both a 4’-amino and 1’4’-imino tautomer in several different biological systems[11,12] (Supplementary Fig. 13a). Tautomerization between 4’-amino and 1’4’-imino states of aminopyrimidine rings has been reported to by catalysed by acetic acid at room temperature[12]. We are aware of no studies of aminopyrimidyl tautomers of thiamine in OCT1 or similar systems, and so cannot exclude possibility of 1’4’-iminopyramidyl thiamine during transport. As such, we have chosen to conduct molecular dynamics simulations with both the 4’-amino and 1’4’-imino tautomers of thiamine.

References

1. Khanppnavar, B. *et al.* Structural basis of organic cation transporter-3 inhibition. *Nature Communications 2022 13:1* **13**, 1–13 (2022).
2. Pymol ScrIpt COllection (PSICO). https://github.com/speleo3/pymol-psico.
3. Punjani, A., Zhang, H. & Fleet, D. J. Non-uniform refinement: adaptive regularization improves single-particle cryo-EM reconstruction. *Nature Methods 2020 17:12* **17**, 1214–1221 (2020).
4. Emsley, P., Lohkamp, B., Scott, W. G. & Cowtan, K. Features and development of *Coot*. *Acta Crystallogr D Biol Crystallogr* **66**, 486–501 (2010).
5. Larkin, M. A. *et al.* Clustal W and Clustal X version 2.0. *Bioinformatics* **23**, 2947–2948 (2007).
6. Waterhouse, A. M., Procter, J. B., Martin, D. M. A., Clamp, M. & Barton, G. J. Jalview Version 2—a multiple sequence alignment editor and analysis workbench. *Bioinformatics* **25**, 1189–1191 (2009).
7. Jumper, J. *et al.* Highly accurate protein structure prediction with AlphaFold. *Nature* (2021) doi:10.1038/S41586-021-03819-2.
8. Waterhouse, A. *et al.* SWISS-MODEL: homology modelling of protein structures and complexes. *Nucleic Acids Res* **46**, W296–W303 (2018).
9. Goddard, T. D. *et al.* UCSF ChimeraX: Meeting modern challenges in visualization and analysis. *Protein Science* **27**, 14–25 (2018).
10. Hernández, B., Pflüger, F., Kruglik, S. G., Cohen, R. & Ghomi, M. Protonation–deprotonation and structural dynamics of antidiabetic drug metformin. *J Pharm Biomed Anal* **114**, 42–48 (2015).
11. Baykal, A. T., Kakalis, L. & Jordan, F. Electronic and nuclear magnetic resonance spectroscopic features of the 1′,4′-iminopyrimidine tautomeric form of thiamin diphosphate, a novel intermediate on enzymes requiring this coenzyme. *Biochemistry* **45**, 7522–7528 (2006).
12. Kitamura, T., Okita, M., Sasaki, Y., Ishikawa, H. & Fujimoto, A. Amino–imino tautomerization reaction of the 4-aminopyrimidine/acetic acid system. *Spectrochim Acta A Mol Biomol Spectrosc* **69**, 350–360 (2008).
