# Supplementary material for: Structural basis of promiscuous substrate transport by Organic Cation Transporter 1
Source: Nat Commun. 2023 Oct 11;14:6374. doi: 10.1038/s41467-023-42086-9 (PMC10567722; doi:10.1038/s41467-023-42086-9)
Supplement: Supplementary file 3 — Description of Additional Supplementary Files [file 41467_2023_42086_MOESM3_ESM.pdf]

Description of Additional Supplementary Files

File Name: Supplementary Movie 1
Description: **Morph between outward-open $OCT1_{CS}$ and inward-open OCT1 and interactions around acidic residues E386 and D474.**

File Name: Supplementary Movie 2
Description: **Representative 200 ns window of MD simulations of metformin and OCT1 showing metformin has sufficient space inside the binding site of OCT1 to reorient.** The Nterminal lobe of OCT1 shown as blue surface, ECD shown in green, TM8 shown in orange. Residue D474 (left) and E386 (right) shown as orange sticks. Phosphate of POPC indicated by grey spheres.

File Name: Supplementary Movie 3
Description: **Representative 200 ns window of MD simulation of metformin and OCT1 showing substrate release.** The N-terminal lobe of OCT1 shown as blue surface, ECD shown in green, TM8 shown in orange. Residue D474 (left) and E386 (right) shown as orange sticks. Phosphate of POPC indicated by grey spheres.

File Name: Supplementary Movie 4
Description: **Representative 200ns window of MD simulation of metformin and OCT1 showing substrate diffusion from E386 to D474.** The N-terminal lobe of OCT1 shown as blue surface, ECD shown in green, TM8 shown in orange. Residue D474 (left) and E386 (right) shown as orange sticks. Phosphate of POPC indicated by grey spheres.
