# Supplementary figures and images for: Structural basis of promiscuous substrate transport by Organic Cation Transporter 1
Source: Nat Commun. 2023 Oct 11;14:6374. doi: 10.1038/s41467-023-42086-9 (PMC10567722; doi:10.1038/s41467-023-42086-9)

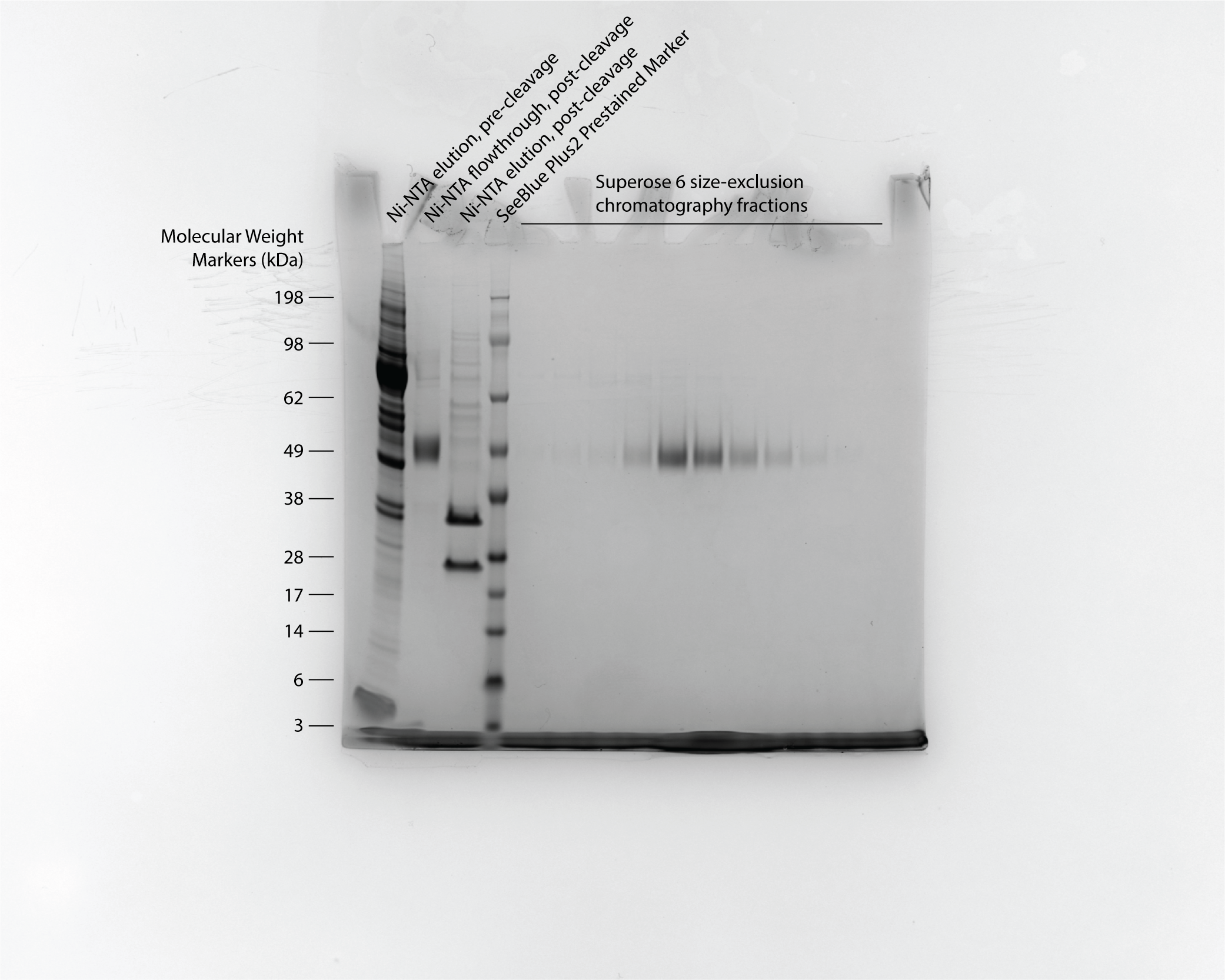

Supplement: Supplementary file 9 — Source Data [file 41467_2023_42086_MOESM9_ESM.zip › Source data/uncropped gel.png]
